# Molecular signaling in coinfection: how *M. tuberculosis* and respiratory viruses rewire host immunity and alter TB outcomes

**DOI:** 10.3389/fimmu.2026.1863302

**Published:** 2026-07-02

**Authors:** Qaqamba Feya, Zilungile Lynette Mkhize-Kwitshana, Ridwaan Nazeer Milase, Ahmed Wadee, Natasha Naidoo, Mamoudou Maiga, Sibusiso Senzani, Lydia Nakiyingi, Nontobeko Eunice Mvubu

**Affiliations:** 1 School of Laboratory Medicine and Medical Sciences, College of Health Sciences, University of KwaZulu-Natal, Durban, South Africa; 2 Biomedical Sciences, Department of Life and Consumer Sciences, College of Agriculture and Environmental Sciences, University of South Africa (UNISA), Florida Science Campus, Johannesburg, South Africa; 3 Research Capacity Development Division, South African Medical Research Council, Cape Town, South Africa; 4 Department of Biochemistry, North-West University, Mahikeng Campus, Mmabatho, South Africa; 5 ThornTree Academic Coaching (PTY) LTD, Johannesburg, South Africa; 6 Climate and Health Research Programme, Environment and Health Research Unit, South African Medical Research Council, Durban, South Africa; 7 Department of Preventive Medicine, Feinberg School of Medicine, Northwestern University, Chicago, IL, United States; 8 Department of Internal Medicine, College of Health Sciences, Makerere University, Kampala, Uganda; 9 Department of Medical Biosciences, Faculty of Natural Sciences, University of the Western Cape, Cape Town, South Africa

**Keywords:** coinfection, immune evasion, inflammation, *Mycobacterium tuberculosis*, pathogen-associated molecular patterns (PAMPs), pattern recognition receptors (PRRs), respiratory viruses, signaling pathways

## Abstract

Tuberculosis (TB) caused by *Mycobacterium tuberculosis* (*M. tuberculosis*) and respiratory viral infections remain major, intersecting global health challenges, and their co-occurrence imposes a disproportionate burden in high-HIV/high-TB regions such as sub-Saharan Africa. Coinfection biology is heterogeneous and dynamic, driven by viral diversity including severe acute respiratory syndrome coronavirus 2 (SARS-CoV-2), influenza A/B, Respiratory Syncytial Virus (RSV), parainfluenza, metapneumovirus, rhinovirus, adenovirus, and bocavirus, and by the underlying TB stage, from latent and subclinical to active and reactivation disease. Innate sensing pathways, such as Toll-like receptors (TLR), retinoic acid-inducible gene I (RIG-I), and cyclic GMP-AMP synthase-stimulator of interferon genes (cGAS–STING), converge during coinfection, reshaping type I interferon (IFN-I), Nuclear Factor kappa-light-chain-enhancer of activated B cells (NF-κB), and AP-1–driven responses and triggering a network of autocrine and paracrine signaling that reprograms macrophages, dendritic cells, and T-cell subsets. This immune rewiring alters granuloma equilibrium through suppressed Th1/IFN-γ coordination, exaggerated Th17/IL-17-driven neutrophilia, and regulatory T-cell or IL-10-mediated dampening, which together destabilize macrophage activation and tissue architecture. Oxidative stress, mitochondrial dysfunction, and Matrix Metalloproteinases (MMP)-driven matrix remodeling further integrate with these pathways, converting inflammatory signals into epithelial damage, cavitation, and fibrosis. Consequently, disease outcomes depend critically on timing, viral burden, pathogen order, host immune endotype, and TB stage, such that the same virus can either preserve containment or drive progression depending on the local immunological context. Importantly, the effects of respiratory viral coinfection vary across the TB disease continuum, influencing early granuloma formation, latent infection, reactivation risk, and established disease through distinct immunological mechanisms. Host-directed therapies (HDT) targeting interferon, IL-1, TNF, inflammasome, or metabolic checkpoints hold mechanistic promise but exhibit variable clinical translation, underscoring the need for precision approaches that integrate stage- and endotype-specific biomarkers. This narrative review proposes an integrated systems framework that links viral sensing, immune rewiring, granuloma biology, and tissue-remodeling to TB–respiratory virus coinfection, and emphasizes how timing-aware, biomarker-guided strategies can refine diagnosis, clinical management, prognosis, and vaccine design in vulnerable populations.

## Graphical Abstract

**Figure:**
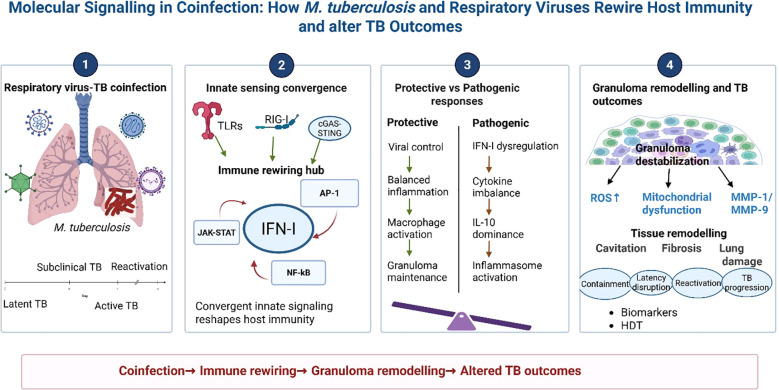

## 1 Introduction

Tuberculosis (TB), caused by *Mycobacterium tuberculosis* (*M. tuberculosis*), remains a major global public health problem, and progress in reducing the burden of disease falls far short of 2030 targets in most parts of the world. In 2024, an estimated 10.7 million people globally developed TB, and approximately 1.23 million deaths were attributed to the disease, with its burden borne disproportionately by low- and middle-income countries (1). In recent years, respiratory viral infections, including influenza, severe acute respiratory syndrome coronavirus 2 (SARS-CoV-2), and others, have introduced additional pressures that further complicate TB control (2). The TB epidemic, both in isolation and when occurring alongside respiratory viral coinfections, is strongly influenced by broad global health determinants, including socioeconomic, biomedical, ecological, and health system factors (3, 4). Respiratory viruses comprise a broad range of molecularly distinct pathogens, including SARS-CoV-2, Parainfluenza virus, Metapneumovirus, respiratory syncytial virus (RSV), Rhinovirus, Adenovirus, Bocavirus, Influenza A, and Influenza B, and acute lower respiratory infections remain leading causes of global mortality and substantial health service burden (5).

Epidemiological evidence indicates that respiratory viruses are frequently detected in TB patients and are associated with increased hospitalization, higher mortality, and greater disease severity across multiple settings. Many of these infections are attributable to respiratory viral pathogens such as RSV, influenza viruses, and coronaviruses, with RSV alone causing about 100–000 global deaths annually in children under five each year (6, 7). Viral coinfection has been linked to reactivation of latent TB and accelerated progression to active disease, particularly in high-risk populations such as those living with HIV, people in sub-Saharan Africa and Southeast Asia, and individuals with socioeconomic deprivation, malnutrition, or diabetes (8). TB-viral coinfections are increasingly recognized as significant contributors to poorer clinical outcomes and rising TB-associated morbidity and mortality worldwide (9).

A central biological challenge in this field is the temporal mismatch between acute, often transient viral infections and chronic, persistent TB infection. Although respiratory viral infections are often transient, they may induce temporally significant perturbations capable of disrupting the finely balanced immune equilibrium required for TB containment (10). Virus-induced changes in innate immunity and epithelial barriers may weaken antimycobacterial defenses and/or exacerbate inflammation, influencing TB progression and outcomes (11). Persistent lung inflammation and structural damage from TB, combined with altered immune responses, further create a permissive environment for viral infections and have been associated with greater disease severity in respiratory viral co-infections such as COVID-19 (12–14).

This narrative review synthesizes recent evidence on the immunological and pathophysiological intersections between *M. tuberculosis* and respiratory viruses, with a systems-level focus on how viral sensing, immune rewiring, granuloma dynamics, oxidative stress, metabolism, and cell death converge to shape inflammatory outcomes (15, 16). We emphasize how timing, TB stage, and host immune endotypes modulate coinfection outcomes, and discuss implications for biomarker development, precision host-directed therapies, diagnosis, clinical management, prognosis, and vaccine design in vulnerable populations (17–19).

## 2 Defining TB-respiratory viral coinfection

TB and respiratory viral coinfection refer to the simultaneous (or interoccurrence) presence of *M. tuberculosis* and the respiratory viruses, which are mainly influenza A/B, SARS-CoV-2, RSV, rhinoviruses, and others, in the same hosts through immunological interaction resulting in differences in disease course, clinical picture, and treatment outcome (2). But for this coinfection model, in addition to mere coexistence of two pathogens, there are three temporal connotations, simultaneous coinfection with both pathogens detected within 7 days, viral first superinfection with the respiratory virus, preceding *M. tuberculosis* activation within 30 days, and TB first superinfection with active TB, preceding the acquisition of the virus (10). Indeed, TB-viral coinfection may be clinically important. In the latest mortality data, TB-influenza coinfection raises the risk of death by 2.3-fold with an adjusted odds ratio of 2.3 and 95% uncertainty interval of 1.02–5.2. In the same manner, TB-SARS-CoV-2 coinfections lead to in-hospital deaths for up to 22.5% cases in low- and middle-income countries, a number that is much higher than for any one infection (20). Globally, there were 10.8 million new cases of TB announced in 2023. Viral coinfections are becoming more widely recognized as critical modulators for TB immunopathogenesis.

Definitions of TB-respiratory viral coinfection have several dimensions. In addition, the temporal window defines a 30-day window for concurrent infections, or a 90-day window for sequential detection of pathogens. The immunological criterion consists of documented type I interferon dysregulation or impairment of *M. tuberculosis*-specific Th1 response that can be established by cytokine profiling and T-cell functional assays (18). Clinical criteria include aggravated respiratory symptoms, more than infection, or increased bacterial and viral load relative to mono-infections. Radiologically, coinfection has overlapping patterns of upper lobe cavitation from TB with bilateral ground-glass opacities attributed to viral pneumonia that may need to be interpreted with clinical expertise for an accurate diagnosis (2).

### 2.1 Diagnostic heterogeneity

The diagnostic heterogeneity in TB–respiratory viral coinfection can be explained by the overlap of clinical manifestations, differences in diagnostics, and the resource-related detection standards. This heterogeneity occurs in three overlapping areas that complicate clinical management and standardization of research. The first major challenge is the clinical symptoms overlap, since both TB and respiratory viruses cause fever, cough, shortness of breath, and tiredness; therefore, diagnostic ambiguity may occur in influenza or SARS-CoV-2 (in which case TB may not be seen at all for the full spectrum of illnesses) pandemics, when TB is underdiagnosed (17). TB-SARS-CoV-2 coinfection poses a particular challenge in clinical pictures, with a mean death rate of 13.9% associated with delayed recognition and initiation of treatment.

Diagnostic modality divergence poses major hurdles for accurate coinfection diagnosis. TB detection, however, depends on GeneXpert *M. tuberculosis*/RIF Ultra and smear microscopy, both of which take 24–48 hours and exhibit a lower sensitivity in coinfection settings, as smear-negative results occur in 30–50% of cases of viral coinfection. Viral detection by means of RT-PCR or rapid antigen tests yields results in less than 4–24 hours, but is not diagnosed by *M. tuberculosis*. This is asynchronous testing, causing diagnostic delays, because sequential testing is necessary, and TB can be missed on the positive side of viral PCR, which may lead to incomplete treatments. Radiological signatures also complicate the diagnosis, as TB is associated with upper lobe cavitation and pleural effusion, and viral pneumonia with bilateral ground-glass opacities, overlaps, which would also result in a doctor’s interpretation that may not be seen in high-burden settings (2).

In low- and middle-income countries with limited access to nucleic acid amplification tests and advanced imaging, resource-dependent detection bias has led to substantial diagnostic gaps and, therefore, leads to a significant impact on coinfection diagnosis. Indeed, machine learning techniques with 98.32% accuracy in COVID-19 detection from surveillance images are, in general, inaccessible in crowded settings with TB burdens, contributing to diagnostic divides. Additionally, traditional tuberculin skin tests and interferon gamma release assays were found to be ineffective in diagnosing active TB in immunocompromised patients with concurrent viral infection, resulting in false-negative findings that delay prompt treatment.

### 2.2 Biological plausibility

TB-respiratory viral coinfection is now theoretically established to be a candidate for TB through its IFN-I-mediated immunopathogenesis, suppression of Th1 cells, and disturbances in myeloid function, as both experimental models of infection show. IFN-I signaling is the main process to connect viral infection to poor TB control because IFN-I signaling due to viral infection disrupts CXCL9/10 uptake in myeloid resulting in poor pulmonary motility of *M. tuberculosis* -related CD4^+^ T cells (18). This mechanism was proved through *M. tuberculosis*-lymphocytic choriomeningitis virus coinfection models, which induced excessive pulmonary bacterial loads due to viral coinfection with corresponding reduction of *M. tuberculosis*-specific IFN-γ production and hyperinflammation and suppression of IFN-I signaling that rescues *M. tuberculosis*-specific IFN-γ response in a lung immune pathologic state.

The interaction of viral IFN-I and dysregulated Th1 reactions may be seen as a cascading process in which viral IFN-I signaling attenuates CXCL9/10 chemokine secretion from myeloid cells, thereby preventing *M. tuberculosis*-specific CD4^+^ T-cell pulmonary influx, leading to higher *M. tuberculosis* overload and worsening of disease. Influenza A virus mainly disrupts *M. tuberculosis* control through an IFNAR1 modulation pathway, whereby coinfection causes reduced *M. tuberculosis*-specific IFN-γ production, elevated bacterial load, and hyperinflammation, and differential cytokine profiles, such as increasing IFN-γ^+^, IL-17^+^CD4^+^, and IFN-γ^+^, IL-17^-^CD8^+^ cells. This enhanced susceptibility to *M. tuberculosis* is also due to the IL-10 signaling pathway through viral coinfection, whereby suppression of IL-10 receptor signaling lowers bacterial loads in coinfected mice to similar levels to *M. tuberculosis*-only infected animals, providing a promising therapeutic modality (2).

The dysregulation associated with SARS-CoV-2 in TB coinfection manifests itself by decreased total lymphocyte counts and impaired CD4^+^ T-cell responses, which can contribute to the disease’s severity by intensifying immunosuppression. During the stage of severe disease, both pathogens occupy a biological niche in the lower respiratory tract, potentially resulting in enhanced or moderated outcomes depending on the temporal sequence and host immune status (21). The structural lung damage caused by TB, such as cavitation and bronchiectasis, creates anatomical niches for viral entry and persistence into the lung, whereas viral-induced damage of epithelial tissues also promotes the invasion and spread of *M. tuberculosis* (22).

### 2.3 Temporal sequence

The temporal pattern of TB-viral coinfection has a critical effect on immunological pathogenicity and clinical outcome and can be reduced into three sequences with different risk profiles and mechanisms. The viral-first sequence is the highest risk configuration and occurs when respiratory virus infection precedes *M. tuberculosis* activation within 30 days. In this case, the host is at risk for a transition from latent TB infection to active TB-related disease (10). Studies prove that viral infections will increase susceptibility for any potential transition from latent *M. tuberculosis* infection to active TB disease by interaction with IFN-I proteins. IFN-I priming is that under which existing IFN-I signaling enables a permissive myeloid environment to be created before *M. tuberculosis*-specific Th1 cells settle in the lung. Resolved influenza A infection in *M. tuberculosis*-infected mice caused increased pulmonary bacterial load through IFN-I-dependent signaling (21). This sequence is the most dangerous configuration because viral-induced immunosuppression precedes the failure of *M. tuberculosis* containment.

Simultaneous coinfection, defined as the detection of both pathogens within 7 days, has particular challenges, including diagnostic difficulties in overlapping symptoms resulting in misdiagnosis and simultaneous immune dysregulation with concurrent IFN-I from viral infection and TNF-α/IL-1β from *M. tuberculosis* infection, leading to potential inflammatory storm (20). This has a considerable mortality burden, with TB-influenza simultaneous coinfection associated with a 2.3-fold increased risk of death and TB-SARS-CoV-2 coinfection presenting with in-hospital fatality rates as high as 22.5% in low- and middle-income countries. The TB-first sequence, in which TB is active and viral infection occurs within 90 days, leads to existing lung damage from TB that increases the risk of acute viral pneumonia, TB-induced immune exhaustion that induces impaired viral clearance, and continued hospitalization associated with increased treatment costs (23).

Immunological outcomes related to each time sequence are conceptually characterized in separate pathways. During the viral-first sequence, there is viral IFN-I signaling that occurs before *M. tuberculosis* exposure, enabling the *M. tuberculosis* replication process to take place before the emergence of adaptive immunity. In simultaneous coinfection, both pathogens simultaneously activate their respective immune pathways, resulting in competing signaling cascades that either are unable to resolve effectively or fail to resolve at all. By contrast, in the TB-first sequence, chronic *M. tuberculosis* infection has already transformed the immune response in its host, and the host cannot efficiently eliminate the viral infection. These temporal relationships are pivotal in the understanding of coinfection pathogenesis and strategies of targeted treatment.

### 2.4 TB stage-specific vulnerability

Vulnerability to respiratory viral coinfection varies between various stages of TB in a highly heterogeneous fashion, with different immunological and structural characteristics at each stage affecting susceptibility to and recovery from coinfection. Under normal circumstances, about 5% of the individuals with latent TB infection will progress to active TB within 2 years; however, respiratory virus infection elevates the risk of latent TB infection to active TB transition by IFN-I-mediated Th1 impairment (10). It involves viral IFN-I signaling that inhibits *M. tuberculosis*-specific CD4^+^ T-cell pulmonary localization before localized granuloma formation is formed, allowing for a crucial window of opportunity in which viral infection can shift patient control toward an outcome of active TB (18).

The highest vulnerability period is early active TB that occurs within the first 2 years after infection because progression from latent to active TB occurs most commonly during this timeframe. Structural properties such as early cavitation provide niches for viral-bacterial co-localization, and incomplete polarization of Th1 can render the host susceptible to viral superinfection. In treatment-naïve active TB, several vulnerability factors converge. Depletion of CD4^+^ T cells has resulted in impaired viral clearance, lung architecture is compromised with increased opportunity for entry, chronic inflammation increases risk of cytokine storms, malnutrition is common in low- and middle-income countries, and worsens immune function (22).

Additional exposures arise in the treatment periods of TB, such as drug interactions involving rifampicin, where CYP3A4 induction may alter antiviral pharmacokinetics, and immune reconstitution, where early ART commencement in HIV-TB-viral triple coinfection elevates immune reconstitution inflammatory syndrome risk, while prolonged exposure since the duration of treatment of six months can lead to nosocomial viral exposure risk (2). Post-treatment TB, whether cured or recurred, presents lung damage that is fibrosis and bronchiectasis, which can further predispose to severe viral pneumonia, and viral infection may induce *M. tuberculosis* recrudescence via reactivation mechanisms (22).

Stage-specific risk can be ascertained associated with different TB stages including latent TB infection, which results in 1.5–2.0–fold increased risk of progression to active TB (IFN-I-mediated Th1 impairment), early active TB (2.3–4.5–fold increased mortality with influenza due to incomplete immunity plus cavitation), established active TB (3.0–fold increased mortality with SARS-CoV-2 due to compromised CD4^+^ cells and structural damage), and treatment-phase TB (1.8–fold increased prolonged hospitalization due to drug interactions plus nosocomial exposure). The quantified risks underscore the necessary considerations regarding the phase of coinfection, as well as the time-sensitive windows for any intervention (10, 20).

## 3 Host recognition, immune sensing, and early immune perturbation

The initial detection of *M. tuberculosis* and respiratory viruses by the host innate immune system determines the trajectory of infection, inflammation, and subsequent adaptive immunity. Pattern recognition receptors (PRRs) serve as the primary sensors that detect pathogen-associated molecular patterns (PAMPs), initiating signaling cascades that shape cytokine networks, cell death pathways, and immune cell recruitment. In coinfection, overlapping or competing PRR activation can rewire host responses, leading to altered disease outcomes (24).

### 3.1 PRR recognition and pathogen sensing

Pathogen recognition starts with attachment to permissive host cells and detection of PAMPs by host PRRs. Although *M. tuberculosis* and respiratory viruses have considerable differences in PAMP composition and PRR engagement, they ultimately share immune evasion strategies. *M. tuberculosis* uses lipid-based PAMPs such as trehalose dimycolate, mycolic acids, and lipoarabinomannan (LAM) for detection. Toll-like receptors (TLRs), NOD-like receptors (NLRs), C-type lectin receptors (CLRs), and the cGAS-STING system detect lipids, proteins, and nucleic acids, primarily due to their recognition of these PAMPs (25, 26). Attachment successfully stimulates phagocytosis by alveolar macrophages to begin entry into the intracellular niche. In the phagosome, *M. tuberculosis* alters phagosome maturation to achieve a protected intracellular niche. In this compartment, *M. tuberculosis* may disrupt phagosome maturation and antigen presentation, favoring intracellular survival and persistence (27, 28).

Respiratory viruses, including SARS-CoV-2, influenza A/B, RSV, rhinovirus, adenovirus, metapneumovirus, parainfluenza, and bocavirus, use glycoproteins and protein-based PAMPs for PRR detection (29). Viral sensing takes place through TLRs, RIG-I-like receptors (RLRs), and NLRs that sense viral RNA or DNA during attachment and entry. Viral replication happens in the cytoplasm or nucleus, depending on viral family, with tightly regulated cycles following entry. Early detection triggers antiviral defense programs and subsequent innate immune signaling pathways (30). The divergent PAMPs and entry modes of *M. tuberculosis* and respiratory viruses suggest similarities of immune evasion mechanisms between these pathogens, such as modulation of PRR signaling as well as interference with host antimicrobial responses (31). These interlocking host-pathogen interactions illustrate shared vulnerabilities in innate immune defense mechanisms, which can be disturbed under conditions of coinfection.

### 3.2 RNA sensors: RIG-I and MDA5 in viral and *M. tuberculosis* immunity

RIG-I and Melanoma Differentiation-Associated protein 5 (MDA5) are cytosolic pattern-recognition receptors that detect viral RNA replication intermediates, with RIG-I recognizing 5′-triphosphate or 5′-diphosphate RNAs enriched in poly-U/UC motifs and MDA5 sensing long double-stranded RNA molecules. Upon activation, both receptors signal through the mitochondrial antiviral signaling protein (MAVS), leading to activation of the TBK1–IRF3 pathway and subsequent induction of IFN-I and antiviral gene programs (32). This signaling axis plays a critical protective role during viral infection by promoting the expression of IFN-I and interferon-stimulated genes (ISGs), which suppress viral replication and establish an antiviral state in infected and neighboring cells (33).

*M. tuberculosis* RNA released into the cytosol via ESX-1 is also sensed by RIG-I and MDA5 but produces paradoxical effects, RLR-driven IFN-I responses associated with increased mycobacterial virulence and impaired bacterial control. A 2023 showed MDA5 deficiency improved control of *M. tuberculosis* and reduced pathology, indicating MDA5-mediated IFN-I can be exploited by *M. tuberculosis* (34). These findings suggest that RNA-sensing pathways may exert pathogen-dependent effects during coinfection, functioning as essential antiviral defenses while simultaneously promoting IFN-I-associated immune environments permissive for *M. tuberculosis* persistence (35).

### 3.3 PRR sensing and signaling crosstalk

During *M. tuberculosis*-respiratory virus coinfection, pattern recognition receptors do not operate as isolated sensors but instead engage in extensive signaling crosstalk that can amplify, dampen, or dysregulate downstream inflammatory responses. Convergent PRR activation during coinfection may amplify or dysregulate downstream inflammatory signaling through overlapping TBK1, NF-κB, and IRF pathways (36). This signaling integration creates a complex network where viral PAMPs can dominate early immune signaling, potentially suppressing or rewiring responses to *M. tuberculosis* and altering disease outcomes (24).

The downstream signaling architecture of PRRs reveals extensive overlap that facilitates cross-regulation during coinfection. TLR3, TLR4, TLR7, and TLR8 all converge on TBK1 and IKKϵ kinases, which phosphorylate IRF3 and IRF7 to drive IFN-I production (24). Similarly, RLRs (RIG-I and MDA5) signal through the mitochondrial adaptor MAVS, which recruits TBK1 and IKKϵ, creating a shared signaling node with TLR3/4/7/8. The cGAS-STING pathway also converges on TBK1-IRF3, establishing a third input into the same IFN-I axis (37). Consequently, simultaneous sensing of viral RNA and *M. tuberculosis*-derived nucleic acids may amplify TBK1-dependent IFN-I signaling, a response associated with virulent *M. tuberculosis* infection and impaired antibacterial immunity (38).

Signaling interference between PRR pathways can occur through multiple mechanisms. NLRX1, a mitochondrial NLR family member, negatively regulates TLR-induced NF-κB signaling by targeting TRAF6 and IKK, potentially dampening antibacterial responses during viral infection. Conversely, STING can directly transmit RIG-I-MAVS-mediated signals, as STING deficiency significantly impairs RIG-I-dependent IFN production even in the absence of DNA sensing (39). This physical interaction between MAVS and STING creates a potentiating loop where viral RNA sensing enhances DNA sensing outputs, and vice versa. During coinfection, this cross-amplification could lead to exaggerated IFN-I responses that suppress protective immunity to *M. tuberculosis*. Cross-regulation also occurs at the transcriptional level, where viral RNA sensing can upregulate STING expression while DNA sensing pathways enhance RLR responsiveness, creating positive feedback loops that sustain IFN-I signaling (36).

CLR, such as Dectin-2 and Mincle, which recognize *M. tuberculosis* mannose cap LAM and cord factors, respectively, cooperate with TLRs to amplify inflammatory signaling through synergistic NF-κB activation (40). However, during viral coinfection, the dominant IFN-I signature driven by TLR7/8 and RLRs can suppress CLR-TLR cooperative signaling, reducing IL-1β and IL-17 production essential for granuloma formation and *M. tuberculosis* control (41). Thus, antiviral PRR dominance during coinfection may suppress CLR-TLR cooperative inflammatory signaling required for optimal anti-mycobacterial immunity.

The inflammasome network introduces an additional layer of cross-regulation. Viral activation of NLRP3 may occur concurrently with IFN-I-mediated suppression of inflammasome maturation, generating dysregulated IL-1β responses that impair antibacterial immunity during coinfection (42, 43).

**Figure 1:**
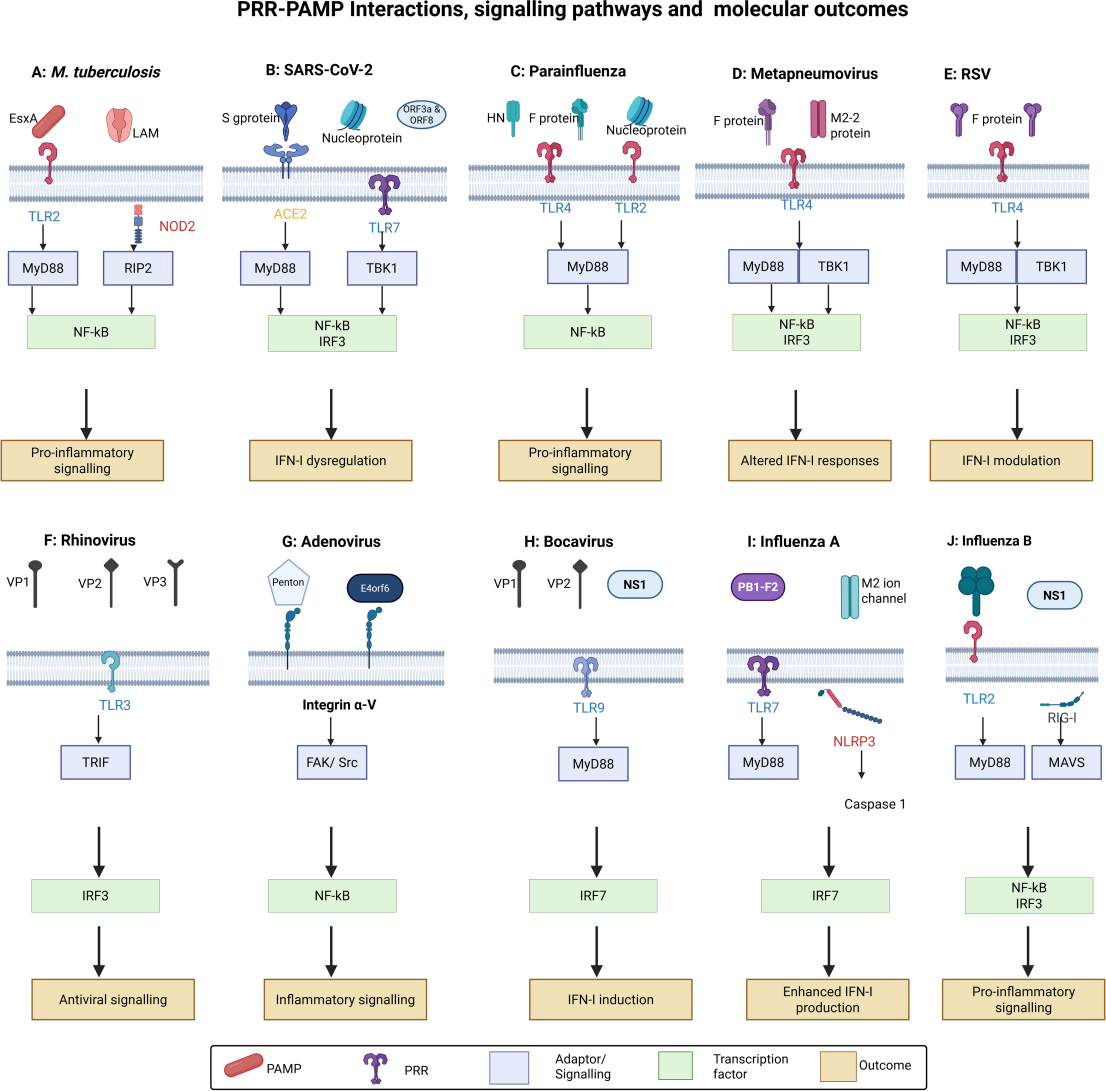
Pattern recognition receptor (PRR)-pathogen-associated molecular pattern (PAMP) recognition and signaling in alveolar macrophages during *M. tuberculosis* and respiratory viral (RV) infection. The image illustrates how *M. tuberculosis* components (e.g., LAM, EsxA) and respiratory viral proteins (e.g., SARS-CoV-2 S and nucleoproteins, influenza and parainfluenza proteins, RSV factors) are recognized by macrophage PRRs, including NOD2, DC-SIGN, TLR2, TLR3, TLR4, TLR7, TLR9, RIG-I, and NLRP3. Engagement of these receptors activates downstream signaling pathways mediated by MyD88, TRIF, MAVS, RIP2, MAPK, NF-κB, and IRF transcription factors. These cascades drive the production of type I interferons, pro-inflammatory cytokines (e.g., IL-6, TNF, IL-1β), inflammasome activation, pyroptosis, and immunomodulatory responses such as IL-10 induction and altered autophagy. Viral and bacterial virulence factors can also subvert these pathways to promote immune evasion. Collectively, *M. tuberculosis* - Respiratory Viral co-recognition results in amplified inflammation, interferon responses, and dysregulated host immunity that may contribute to disease severity.

**Table 1** summarizes the molecular interactions between *M. tuberculosis* and key respiratory viruses, focusing on the host immune system, including PAMPs or attachment factors, host receptors, and PRRs, as well as intracellular replication niches, modes of egress, and immune evasion strategies. *M. tuberculosis* is distinguished by its lipid-rich cell wall, which engages multiple innate immune receptors, including TLRs, CLRs, and cytosolic DNA sensors (26), enabling survival within macrophage phagosomes and long-term persistence through granuloma formation. Its immune evasion strategies extend beyond interferon antagonism to include inhibition of phagosome-lysosome fusion, suppression of antigen presentation, altered cytokine production, and induction of regulatory T-cell responses (16). In contrast, respiratory viruses such as SARS-CoV-2, influenza viruses, and paramyxoviruses employ surface glycoproteins to bind host cell receptors and replicate predominantly in cytoplasmic or nuclear compartments of epithelial cells. Viral dissemination typically occurs through budding, exocytosis, syncytium formation, or host cell lysis (44–46). Despite differences in replication strategies, a unifying feature among these viruses is the capacity to blunt innate immune sensing, particularly interferon signaling, through the action of non-structural proteins (NSPs), modulation of PRR pathways, or compartmentalization of viral replication. Several viruses further enhance persistence or transmission through antigenic variation, immune cell recruitment modulation, or delayed apoptosis (47). Together, this comparison highlights how bacterial and viral respiratory pathogens converge on shared immune escape pathways while maintaining distinct replication niches and survival strategies that influence disease severity, persistence, and host-pathogen dynamics (**Table 1**; **Figure 1**).

**Table 1:** Comparative overview of attachment, replication, and immune evasion strategies of *Mycobacterium tuberculosis* and major respiratory viruses.

Pathogen	Key PAMPs/attachment factors	Host receptors/PRRs	Primary replication site	Exit strategy	Major immune evasion mechanisms	TB outcomes	References
*Mycobacterium tuberculosis*	Lipid-based PAMPs (LAM, trehalose dimycolate, mycolic acids)	TLR2, TLR4, mannose receptor, NOD-like receptors, CLRs, cGAS–STING	Phagosome within alveolar macrophages	Host cell necrosis or apoptosis; granuloma-mediated persistence	Inhibition of phagosome-lysosome fusion; suppression of MHC II and cytokine production; induction of regulatory T cells; latency within granulomas	Latent TB infection or active pulmonary TB with granuloma formation and potential reactivation	(16, 115–117)
SARS-CoV-2	Spike (S) protein (S1/S2 subunits)	ACE2; PRRs including RIG-I, TLR7	Cytoplasm (ER–Golgi intermediate compartment)	Exocytosis via Golgi vesicles	Inhibition of interferon signaling (NSP1, ORF6); interference with PRR detection; suppression of host gene expression	Enhanced lung inflammation and impaired antimycobacterial immunity, potentially worsening or reactivating TB	(44, 118–121)
Parainfluenza viruses (HPIV)	Hemagglutinin-neuraminidase (HN), Fusion (F) protein	Sialic acid-containing receptors; RIG-I, TLRs	Cytoplasm	Budding from the plasma membrane	Interferon suppression, modulation of host lipid metabolism, potential persistence	Suppression of Th1 responses may increase susceptibility to TB progression	(45)
Human metapneumovirus (HMPV)	Fusion (F), Glycoprotein (G), SH protein	Sialic acid receptors; RIG-I, TLR7	Cytoplasmic inclusion bodies	Budding	Interference with RIG-I signaling, compartmentalization of replication to evade immune detection	Immune dysregulation may weaken antimycobacterial responses and exacerbate TB pathology	(122, 123)
Respiratory syncytial virus (RSV)	G and F proteins	CX3CR1; TLRs	Cytoplasm	Budding; syncytium formation	NS proteins inhibit interferon responses, immune cell recruitment modulation	Th2-skewed inflammation may suppress protective TB immunity and promote disease progression	(46, 124)
Rhinoviruses	Capsid proteins	ICAM-1, LDLR, CDHR3	Cytoplasm	Host cell lysis	Viral protease-mediated suppression of interferon pathways	Airway inflammation and macrophage dysfunction may increase susceptibility to TB	(125, 126)
Adenoviruses	Fiber knob proteins	CAR, CD46, CD80/CD86	Nucleus	Host cell lysis	Inhibition of apoptosis; suppression of interferon signaling	Immune suppression may reduce host control of *M. tuberculosis*	(127, 128)
Human bocavirus	Capsid and NS proteins	Receptor(s) not fully defined	Nucleus	Host cell lysis	Interference with apoptosis and interferon signaling; episomal persistence	Persistent airway inflammation may increase risk of TB progression	(129–131)
Influenza A & B viruses	Hemagglutinin (HA), Neuraminidase (NA)	Sialic acid-linked glycans; TLRs, RIG-I	Nucleus	Budding from the plasma membrane	NS1-mediated interferon inhibition; antigenic drift	Type I IFN responses may suppress Th1 immunity and promote TB progression or reactivation	(132, 133)

## 4 TB stage-specific immune effects

### 4.1 Latent TB: granuloma equilibrium and IFN-I disruption

In latent TB, granulomas maintain a relatively stable equilibrium in which *M. tuberculosis* persists in a non-replicating or slow-replicating state under sustained immune control. Human and murine studies agree that granulomas in latency are characterized by organized macrophage–lymphocyte aggregates, low-grade IFN-γ signaling, and balanced pro- and anti-inflammatory cytokines that restrain bacterial growth without causing overt tissue destruction. Organoid models of TB granuloma-like structures similarly show steady, IL-12- and IFN-γ-dependent macrophage activation that limits bacterial spread while preserving structural integrity (48, 49).

However, this equilibrium is fragile. Experimental murine models indicate that exogenous IFN-I signaling, for example, from viral infection, may disrupt latent containment by suppressing IL-1/TNF and Th1-supporting cytokines, blunting macrophage microbicidal activity, and altering granuloma cellularity (50). Limited human clinical evidence from TB–COVID-19 cohorts suggests that SARS-CoV-2–driven IFN-I signatures correlate with transient reactivation-like events or increased risk of progression in individuals with presumed latent TB, underscoring that latent infection is not an inert state but a tightly regulated balance susceptible to viral-induced immune rewiring (51). Thus, latent TB represents a state of unstable homeostasis in which viral coinfection can tilt the system toward reactivation risk without immediately manifesting as radiographic or clinical disease.

### 4.2 Subclinical TB: inflammatory transition state and unstable containment

Subclinical TB, often defined as microbiologically or immunologically confirmed *M. tuberculosis* infection without classic symptoms, reflects an inflammatory transition state in which bacterial containment is loosening but overt caseation or cavitary disease is not yet apparent (52). Human cohort data show that subclinical TB is associated with elevated inflammatory markers (for example, IL-6, TNF-α, CRP) and early granuloma remodeling, suggesting that immune containment is becoming unstable. *In vitro* studies of human macrophages infected with *M. tuberculosis* further indicate that partial failure of IFN-γ–mediated activation or excessive IL-10 signaling can promote intracellular persistence and low-level dissemination, consistent with the “leaky” containment inferred in subclinical disease (53).

In this context, viral coinfection can critically amplify the transition toward active disease. Experimental murine models of sequential TB-virus infection show that IFN-I–biased environments can accelerate the breakdown of subclinical control, with increased bacterial load and granuloma expansion rather than resolution (54). Organoid models of TB-granuloma-like structures subjected to viral mimicry, such as IFN-I stimulation, similarly demonstrate loosening of cell aggregates and reduced bacterial containment, supporting the idea that subclinical TB is an actively changing state of immune balance (50). Therefore, subclinical TB should be viewed not as a static pre-TB phase but as a dynamic inflammatory transition state where viral-driven immune rewiring can determine whether containment stabilizes or progresses.

### 4.3 Active TB: hyperinflammation, ROS, MMP activation, and tissue destruction

Active pulmonary TB is characterized by hyperinflammation, granuloma necrosis, and lung tissue destruction rather than the balanced containment seen in latent or subclinical TB. Human clinical observations and histopathological studies consistently show enlarged granulomas with central caseation, perigranulomatous inflammation, and upregulation of pro-inflammatory cytokines (TNF-α, IL-1β, chemokines) and lipid-mediated signals that drive tissue remodeling (55). *In vitro* systems using human macrophages or lung epithelial cells exposed to *M. tuberculosis* replicate aspects of this state, revealing strong reactive oxygen species (ROS) production, elevated nitric oxide, and pro-apoptotic and necrotic death pathways that support granuloma breakdown.

A key driver of tissue destruction is the activation of matrix-metalloproteinases (MMPs), particularly MMP-1 and MMP-9, which are markedly upregulated in active TB and associated with matrix degradation and cavity formation (56). Experimental murine models of progressive TB show that MMP-1 overexpression or loss of tissue inhibitors of metalloproteinases (TIMPs) correlates with enhanced lung damage and bacterial dissemination, whereas MMP inhibition can preserve lung architecture despite persistent infection. Organoid-based models incorporating human lung-derived cells and *M. tuberculosis* likewise demonstrate that inflammatory signals, such as TNF-α and IFN-γ, which may induce MMP expression and disrupt epithelial–stromal integrity, recapitulate the matrix-destructive environment of active TB (57). Thus, active TB is not simply “more TB” but a distinct immunological state dominated by hyperinflammation, ROS-driven stress, MMP-mediated matrix degradation, and structural collapse rather than containment.

### 4.4 Reactivation: chronic immune dysregulation and impaired immune coordination

Reactivation TB, whether after a period of latency or following treatment-induced remission, reflects chronic immune dysregulation and impaired coordination of antiviral and antibacterial responses rather than a simple re-emergence of the same latent program. Experimental murine models of TB reactivation, for instance, via immunosuppression, cytokine blockade, or viral co-challenge, show that disrupted Th1/IFN-γ signaling, elevated IL-10, and sustained IFN-I skewing can release *M. tuberculosis* from quiescence and promote granuloma breakdown. *In vitro* human studies further demonstrate that reductions in IFN-γ responsiveness or increased regulatory T-cell activity can impair macrophage microbicidal function and allow bacterial resuscitation, consistent with the “dysregulated equilibrium” proposed in reactivation.

Human clinical evidence from cohorts with HIV, post-transplantation, or persistent viral infections (including SARS-CoV-2) indicates that chronic immune dysregulation, such as low IFN-γ, elevated IL-10, and IFN-I-skewed signatures, predisposes to reactivation TB, often in a pattern of recurrent or difficult-to-contain inflammation rather than stable latent disease (58). Organoid-like systems modeling reactivation-associated conditions, such as cytokine shifts or drug-induced immunomodulation, mirror these features, showing disrupted granuloma architecture and increased bacterial release when Th1/IFN-γ circuits are suppressed (49). Therefore, reactivation is not a reversal to the same latent state but a distinct, dysregulated immunological configuration in which prior containment has failed, and immune coordination is chronically impaired.

Building on the integrated signaling model shown in **Figure 1**, we identify that the coinfection of *M. tuberculosis* with the respiratory virus cannot be sufficiently elucidated by static pathway mapping. Although the major pattern recognition receptors, downstream signaling cascades and effector outputs that are linked to infections are presented in **Figure 1**, they are not temporally uniform or biologically equivalent among disease stages. Instead, the immune effect of coinfection is determined by the timing, duration and sequence of pathway activation, and by the existing state of the host-pathogen interface. The perturbations of acute viral infections are transient and high amplitude, especially in type I interferon, inflammasome and cytokine networks, which can transiently modify granuloma dynamics without causing permanent immune dysfunction. These findings require a departure from pathway-oriented understanding and an extension to a temporal and context-dependent paradigm that considers immune responses as dynamic phenomena that unfold over distinct phases of the infection and host susceptibility.

## 5 Temporal dynamics of immune perturbation during coinfection

Short-lived viral infections can transiently reconfigure the lung and granuloma microenvironment in ways that disproportionally influence the trajectory of chronic TB, even when the virus itself is cleared within days or weeks. The key insight is that the temporal and contextual imprint of acute viral perturbation, rather than permanent immunosuppression, can shift the balance between immune containment and bacterial dissemination, underscoring the importance of timing, viral burden, host immune endotype, and pre-existing TB stage.

### 5.1 Dynamic nature of granuloma equilibrium

Granulomas in TB are not histologically fixed structures but highly dynamic niches that constantly adjust in response to T-cell recruitment, macrophage activation, and local cytokine gradients. Long-term containment of *M. tuberculosis* depends on the maintenance of a “moving equilibrium” in which effector T cells provide ongoing immune surveillance, macrophages oscillate between bactericidal and tissue-remodeling states, and chemokine gradients (for example, CXCL9/10-CXCR3) continuously recruit fresh T-cell reinforcements to the lesion (59, 60).

Recent multimodal profiling of lung granulomas in non-human primates shows that lesions with early, “loose” organization and delayed T-cell influx are associated with higher bacterial burdens, whereas granulomas formed later, after adaptive priming has matured, more frequently achieve containment (18). This temporal plasticity implies that any acute-phase event, including a short viral infection, that delays T-cell recruitment, alters macrophage polarization, or distorts CXCL9/10 gradients, can temporarily destabilize what otherwise appears to be a stable granulomatous equilibrium, thereby creating a permissive window for *M. tuberculosis* expansion even if the overt pathology later resolves (60).

### 5.2 Acute viral immune perturbation

Acute respiratory viruses such as influenza A or lymphocytic choriomeningitis virus (LCMV) trigger large, transient bursts of IFN-I, which rapidly reshape the lung and draining lymph-node compartment (61). Although IFN-I surges are typically self-limiting, they can induce a period of transient immune suppression or dysregulation during which IFN-I-dependent signaling inhibits CXCL9/10 production by myeloid cells and impairs the recruitment of *M. tuberculosis*-specific Th1 cells to the lung, thereby dampening IFN-γ–mediated bacterial control (18).

This acute phase is accompanied by shifting macrophage polarization states, where IFN-I-driven programs can both suppress classical *M. tuberculosis*-directed activation and promote alternative phenotypes linked to tissue repair or neutrophil-rich inflammation (54). Concurrent epithelial injury and local cytokine imbalance, for example, elevated IL-10, IL-4, or chemokines recruiting neutrophils over effector T cells, further skew the granuloma microenvironment toward a more permissive state for *M. tuberculosis* replication, even though the underlying genetic and metabolic architecture of the lesion may remain unchanged (61).

Critically, this acute viral perturbation does not necessarily equate to permanent immune dysfunction; many models show that IFN-I signatures and Th1 deficits can normalize after viral clearance, yet the short-term window of impaired immune surveillance is sufficient to raise *M. tuberculosis* set-points, increase dissemination, and worsen long-term disease severity (62). This distinction, that *acute perturbation* ≠ *permanent immune change*, is central: brief viral episodes can still “reset” the host-pathogen race by altering the timing and location of immune cell arrival, even if the overall immune potential later recovers.

### 5.3 Context-dependent determinants of coinfection outcomes

The impact of acute respiratory viral infection on chronic TB trajectory cannot be reduced to a single outcome but instead emerges from the intersection of viral burden, infection timing relative to *M. tuberculosis* exposure, TB disease stage, and host immune endotype. These variables interact to determine whether transient viral perturbation normalizes after viral clearance or instead establishes a new, less protective immune trajectory that favors *M. tuberculosis* expansion.

Timing is relative to the TB stage. Viral coinfection occurring before or during the critical window of *M. tuberculosis*-specific T-cell priming and granuloma organization can delay antigen transport to draining lymph nodes, impair Th1 differentiation, and disrupt CXCL9/10-CXCR3 gradients that guide effector T-cell recruitment to nascent lesions (18). This early disruption leads to higher bacterial loads, neutrophil-rich pathology, and “loose” granuloma architecture with delayed containment. Conversely, similar viral perturbations occurring after robust granuloma maturation and established Th1 surveillance may produce only modest or transient effects, as the adaptive immune architecture is already in place and less susceptible to acute IFN-I-mediated disruption (24). The phase of TB, early infection versus established latency versus incipient reactivation, therefore, dictates the degree of vulnerability to viral immune perturbation.

Host immune endotype and baseline responsiveness. Individual variation in baseline IFN-I responsiveness, T-cell repertoire diversity, and propensity for type-2 or neutrophilic inflammation determines whether a transient viral episode resolves without lasting impact or instead amplifies into sustained immune dysregulation (41). Hosts with high intrinsic IFN-I responsiveness may experience prolonged IFN-I signaling even after viral clearance, sustaining suppression of CXCL9/10 and Th1 recruitment (18). Pre-existing inflammation, whether from prior TB exposure, HIV coinfection, or metabolic comorbidities, can further amplify viral-induced IFN-I signals and epithelial damage, magnifying the risk of *M. tuberculosis* reactivation or accelerated progression. This heterogeneity suggests that identical viral insults applied to different hosts or different granuloma microenvironments can yield divergent TB outcomes (24).

Viral burden and IFN-I amplitude. The magnitude and duration of IFN-I responses scale with viral replication kinetics and tissue burden. High-dose or prolonged viral infections generate sustained IFN-I signatures that more deeply suppress Th1 immunity and CXCL9/10 production, whereas low-dose or rapidly controlled infections may produce only transient perturbations insufficient to alter *M. tuberculosis* set-points (41). This dose-response relationship implies that even the same viral strain can produce variable TB outcomes depending on inoculum, route of infection, and host antiviral capacity.

Conceptual principles emerging from temporal and contextual analysis. Three principles emerge from the integration of granuloma dynamics and acute viral perturbation. First, context-dependence, identical viral insults yield divergent outcomes depending on host immune endotype, TB disease stage, and lesion maturity (62). Second, temporal dependence, the phase of TB at the time of viral infection dictates vulnerability, with early granuloma formation representing a critical window of susceptibility (50). Third, immune heterogeneity, individual differences in IFN-I signaling amplitude, macrophage polarization bias, and T-cell homing efficiency shape whether brief viral episodes leave persistent marks on TB trajectory or resolve without long-term consequences (61).

These principles suggest that coinfection outcomes are not deterministic but instead reflect the balance between viral-induced perturbation amplitude, TB lesion resilience, and host capacity for immune recalibration after viral clearance (60). Whether transient IFN-I surges permanently shift *M. tuberculosis* burdens or merely delay containment without altering outcomes remains an area where experimental timing studies and longitudinal immune profiling are needed to distinguish acute immune dysfunction from durable reprogramming (49).

### 5.4 Integrative synthesis: timing and immune “reset” hypothesis

Short viral infections can matter in chronic TB not because they irreversibly damage the immune system, but because they generate time- and context-specific disturbances in granuloma equilibrium, T-cell recruitment, and macrophage polarization that transiently favor *M. tuberculosis* establishment or expansion (62). Because granulomas are dynamically regulated structures whose functional output depends on the precise timing and quality of immune signals, even brief perturbations, such as IFN-I surges, epithelial injury, or cytokine imbalances, can alter disease trajectories by reshaping the early immune landscape within which *M. tuberculosis* adapts and persists (60). This reframing underscores that the duration of a viral infection is less predictive of its impact than the phase of TB and the immune context in which the perturbation occurs, making the temporal dynamics of coinfection a central axis for both mechanistic studies and host-directed interventions.

## 6 Temporal and context-dependent immune rewiring

Host immune responses to *M. tuberculosis* are finely balanced to permit bacterial containment while limiting immunopathology; however, this equilibrium can be profoundly altered during respiratory viral coinfection. Importantly, the immunological consequences of coinfection are not static but depend on the timing, sequence, and context of pathogen exposure. Whether viral infection precedes, coincides with, or follows *M. tuberculosis* infection can influence the magnitude and duration of interferon responses, inflammatory signaling, cellular recruitment, and granuloma stability (63). Similarly, outcomes are shaped by viral burden, pathogen-specific immune signatures, host factors such as age and comorbidities, and the stage of tuberculosis infection, including latent, incipient, or active disease. These variables determine how shared signaling networks, including PRRs, interferon pathways, NF-κB/MAPK cascades, inflammasomes, metabolic checkpoints, and adaptive immune programmes, are integrated and ultimately whether immune responses favor pathogen control, bacterial reactivation, hyperinflammation, or tissue damage (64). Understanding this temporal and context-dependent immune rewiring is essential for explaining the marked heterogeneity observed in clinical coinfection outcomes and for identifying opportunities for precision host-directed therapeutic interventions.

### 6.1 The IFN-I paradox and immune coordination

IFN−I are the conceptual anchor of coinfection−driven immune rewiring because they bridge antiviral defense, inflammation, granuloma biology, cytokine balance, and cellular activation states (10). During respiratory viral infections, IFN−I and, in some settings, IFN-III are essential for limiting replication by inducing antiviral genes, enhancing antigen presentation, and priming cytotoxic and NK−cell responses. However, the magnitude, timing, and persistence of IFN−I vary among respiratory viruses. SARS−CoV−2 and influenza A/B often provoke robust or dysregulated IFN−I responses; RSV and human metapneumovirus can drive strong IFN−I in severe disease or in infants, whereas rhinovirus typically elicits more localized, transient IFN−I signaling (65). These virus−specific IFN−I signatures shape how antiviral responses intersect with antibacterial immunity to *M. tuberculosis*.

When IFN−I signaling is prolonged or excessive during coinfection, it can undermine host control of *M. tuberculosis* at multiple levels. In TB and TB–COVID−19 coinfection, elevated IFN−I signaling correlates with increased bacterial load and impaired containment, suggesting that sustained IFN−I suppresses protective antibacterial pathways (66). Mechanistically, IFN−I suppresses IL−1-dependent inflammatory pathways, inhibits IL−1–driven recruitment and activation of innate effectors that constrain bacterial replication, blunts IL−12–driven Th1 differentiation, and reduces IFN−γ production by CD4 T cells and ILCs, weakening the core axis for macrophage activation and intracellular killing (67). Influenza-driven IFNα/β receptor signaling has additionally been shown to impair alveolar macrophage phagocytosis of *M. tuberculosis* through reduced ROS generation and diminished inducible nitric oxide synthase (iNOS) activity (41). IFN-I signaling may also downregulate chemokines such as CCL2 and CCL5, limiting recruitment of protective T cells to infected tissues and creating a permissive niche for bacterial expansion (21). Beyond innate immune dysfunction, persistent interferon signaling reshapes adaptive immunity. Repeated IFN-I exposure can reduce MHC-II expression on dendritic cells, impair antigen presentation, and weaken priming of *M. tuberculosis*-specific T-cell responses (64). In macrophages, IFN−I skews activation away from classical bactericidal phenotypes, reducing phagosome maturation and antimicrobial effector mechanisms, while sometimes inducing regulatory molecules that favor bacterial persistence (10). Collectively, these changes destabilize granulomas, loosening the multicellular architecture that contains *M. tuberculosis* and promoting dissemination and tissue pathology.

Timing and context are decisive. A short, early IFN−I pulse, typical of controlled rhinovirus infection, may provide antiviral defenses without long−term disruption of antibacterial pathways. In contrast, high viral burden or persistent IFN−I signaling, seen with severe SARS−CoV−2, certain influenza strains, or prolonged RSV/human metapneumovirus infections, can sustain suppression of IL−1β/TNF−α and Th1 axes, tipping the balance toward impaired *M. tuberculosis* control (65). Host factors, including age, immune endotype, comorbidities, and *M. tuberculosis* disease stage (latent versus active), further influence whether IFN−I’s net effect is protective or pathogenic. Thus, IFN−I links antiviral immunity, inflammation, granuloma integrity, and cytokine imbalance to produce the immune rewiring observed during coinfection (66).

Experimental evidence further indicates that the order of infection is an important determinant of outcome. Viral infection preceding recruitment of *M. tuberculosis*-specific Th1 cells can exacerbate disease through IFN-I-dependent impairment of pulmonary Th1 trafficking. In addition to suppressing macrophage antimicrobial functions, IFN-I reshapes antigen presentation and chemokine gradients required for effective Th1 localization. These findings suggest that the immunological consequences of virus→TB and TB→virus coinfection may differ substantially and should be considered when interpreting both experimental and clinical studies (18).

### 6.2 Cytokine and inflammatory crosstalk

Coinfection reorganizes immunity through dynamic interaction among IL−1β, TNF−α, IL−10, IFN−γ, TGF-β, and IFN−I rather than through isolated cytokine changes. Respiratory viruses differ in how they shift this balance: influenza A/B and SARS−CoV−2 often elicit strong systemic IFN−I and downstream regulatory cytokine responses that may suppress IL−1β/TNF−α pathways, whereas rhinovirus typically induces a more mucosal, transient profile (67). In TB–COVID−19 coinfection, elevated IFN−I and IL−10 relative to uncomplicated TB are associated with impaired Th1 function and reduced granuloma−supporting cytokine networks (65).

At the molecular level, these cytokine changes are coordinated through activation of the NF-κB and MAPK signaling pathways, which serve as central regulators of inflammatory gene expression (68). Integration of mycobacterial and viral signals activates NF-κB-dependent transcription of cytokines, chemokines, and adhesion molecules, while MAPK pathways (p38, ERK, and JNK) amplify cytokine production and cellular stress responses (69). *M. tuberculosis* components such as lipoarabinomannan engage TLR2-MAPK signaling, whereas viral sensing through RIG-I and related pathways enhances ERK-dependent production of CXCL8 and CXCL10, promoting recruitment of neutrophils and inflammatory monocytes (23, 68). Under dual stimulation, dysregulated NF-κB/MAPK activation can sustain excessive IL-6 and TNF-α production, driving tissue injury and endothelial dysfunction while simultaneously compromising effective antimicrobial control (70).

When IFN−I dominates, suppression of IL−1β and TNF−α reduces macrophage recruitment and licensing, weakening granuloma maintenance. If IL−1β/TNF−α/IFN−γ responses predominate, antibacterial containment strengthens, but the risk of tissue−damaging inflammation rises (69). Regulatory cytokines, particularly IL−10 and TFG-β, function as critical counterbalances. Virus−induced IL−10 can protect tissues from collateral damage, yet, when overproduced, as reported in severe influenza and SARS−CoV−2, it can blunt Th1 responses and granuloma integrity. The result is nonlinear: modest IL−1β potentiates IL−12/IFN−γ loops and granuloma formation, whereas excessive inflammation in the context of high viral replication drives necrosis and pathology (70). Coinfection therefore threatens the equilibrium between protective containment and pathological hyperinflammation by simultaneously activating antiviral and antibacterial cytokine circuits.

### 6.3 Inflammasome dynamics and inflammatory amplification

Inflammasomes are central regulators of innate immunity that link pathogen sensing to inflammatory amplification through activation of caspase-1 and maturation of IL-1β and IL-18. Among these complexes, NLRP3 and AIM2 are particularly important during *M. tuberculosis*–respiratory viral coinfections, where they function at the intersection of antibacterial defense and inflammation-mediated tissue injury (71). Granuloma destabilization and immune equilibrium NLRP3−driven inflammasome activation and IL−1β maturation are central amplifiers of antibacterial responses, yet they sit in a dynamic regulatory tug−of−war with IFN−I. Many respiratory viruses, including influenza A and SARS−CoV−2, can activate or modulate NLRP3 and IL−1β/IL−18 release, often in a way that exacerbates lung inflammation (66). In *M. tuberculosis* infection, inflammasome−derived IL−1β supports granuloma formation and macrophage recruitment, but IFN−I signaling can suppress inflammasome activity and IL−1β maturation, thereby reducing a critical amplifier for bacterial containment.

Conversely, uncontrolled inflammasome activation drives pyroptosis and releases damage-associated molecular patterns (DAMPs), which in the context of viral−induced tissue injury can amplify inflammation and compromise granuloma integrity. Recent work links plasma-membrane or phagosome damage to NLRP3 activation and pyroptosis during *M. tuberculosis* infection, placing membrane integrity and ion flux at the center of inflammasome biology in TB. SARS-CoV-2 provides complementary mechanisms: viroporins (including ORF3a) can prime NF-κB-dependent IL−1β transcription and activate NLRP3 through ion-channel activity and K+ efflux, thereby converging mechanistically with *M. tuberculosis*-driven danger signals (72–74). AIM2 and NLRP3 may act synergistically to enhance gasdermin D-mediated pore formation, epithelial injury, and bacterial dissemination. Excessive IL-1β production promotes Th17 recruitment, neutrophilic inflammation, and fibrosis, whereas IL-18-induced IFN-γ responses may be insufficient to overcome viral suppression of Th1 immunity (75). Furthermore, crosstalk between caspase-8-dependent apoptotic pathways and inflammasome signaling generates mixed forms of inflammatory cell death that impair efferocytosis and prolong tissue injury (72).

In severe influenza or SARS−CoV−2 coinfection, hyperactive inflammasome signaling has been associated with enhanced IL−1β release and tissue damage, illustrating how inflammasome ↔ IFN−I imbalance can shift from protective antibacterial control to harmful inflammatory amplification. The same virus may tip this balance differently depending on viral load and host context: low−level rhinovirus exposure may allow beneficial IL−1β signaling, whereas high−burden influenza or severe SARS−CoV−2 may either hyper−activate inflammasomes or, via strong IFN−I/IL−10 programs, paradoxically suppress effective IL−1β maturation (71).

### 6.4 Granuloma destabilization and immune equilibrium

Granulomas are dynamic immune structures whose integrity depends on coordinated macrophage function, Th1 recruitment, and balanced cytokine/inflammatory inputs. Viral coinfections that alter IFN-I, IL-1β, TNF-α, and IFN-γ dynamics, especially viruses that provoke sustained systemic IFN-I responses, such as influenza A/B and severe SARS-CoV-2, erode these processes. In TB–COVID-19 coinfection, clinical and immunological data show reduced Th1 cell influx and IFN-γ production, together with elevated IFN-I and IL-10, which are associated with impaired granuloma maintenance and increased bacterial burden (41, 60).

Reduced Th1 recruitment and IFN-γ signaling weaken the adaptive scaffold; dampened IL-1/TNF signaling undermines macrophage microbicidal activation and intercellular adhesion; and altered macrophage phenotypes become more permissive to intracellular growth. Combined with inflammasome-driven pyroptosis or virus-induced tissue injury, these shifts destabilize granulomas, promoting bacterial dissemination and lesion progression (23). Viruses with more localized, transient IFN-I signatures (rhinovirus, some adenoviruses) may produce milder or reversible granuloma perturbations, whereas high-burden or dysregulated infections (severe influenza, RSV in infants, SARS-CoV-2) are more likely to drive sustained destabilization. Framing granulomas as the emergent outcome of cytokine-level rewiring clarifies mechanistic links between molecular signaling and clinical TB outcomes (67).

### 6.5 Context-dependent inflammatory outcomes

Outcomes of viral-*M. tuberculosis* coinfection is conditional rather than deterministic and depends on interacting variables. Pathogen order matters: virus-before-*M. tuberculosis* exposures often prime IFN-I-dominant programs that hinder subsequent antibacterial containment, while virus-after-*M. tuberculosis* can either drive reactivation or exacerbate existing lesions, depending on timing. Viral burden and persistence are critical: high or persistent replication (severe influenza, uncontrolled SARS-CoV-2, prolonged RSV/hMPV) sustains IFN-I and regulatory programs, whereas low-burden or quickly controlled infections (rhinovirus) are less disruptive (67).

TB stage modulates susceptibility: latent and incipient TB have fragile granuloma equilibria that are more sensitive to IFN-I–driven perturbation than robustly contained latent or active TB lesions. Host immune endotype, including polymorphisms affecting IFN-I signaling, inflammasome responsiveness, or IL-1/TNF pathways, shapes how strongly these pathways are engaged (76). Timing and kinetics matter: short intervals between infections favor additive interactions, whereas long intervals allow immune reset or memory shaping that changes outcomes. Immune baseline and comorbidities such as HIV, diabetes, or immunosuppressive therapies modulate thresholds for protective containment versus pathology (77). These factors produce outcomes across a spectrum: preserved control, transient granuloma loosening with recovery, accelerated progression to active disseminated TB, or severe immunopathology, underscoring the need to integrate virus-specific signaling patterns into host-immune stratification strategies.

## 7 Immunometabolism, oxidative stress, and cell death pathways

In *M. tuberculosis*-respiratory−virus coinfection, oxidative stress, metabolic reprogramming, and matrix−remodeling pathways converge with regulated cell death programs to form a mechanistic tissue−injury axis that links inflammatory cytokines to cavitation and fibrosis. Rather than three disconnected modules, metabolism, ROS, and cell death feed into MMP−driven matrix degradation such that the inflammatory signal → metabolic shift → oxidative stress → cell death → tissue remodeling sequence becomes a key driver of pathology.

### 7.1 Metabolic reprogramming, oxidative stress, and tissue injury

Metabolic reprogramming represents a central battleground during *M. tuberculosis*–respiratory viral coinfection because cellular metabolism directly regulates antimicrobial immunity, inflammatory signaling, oxidative stress, and tissue repair. Host responses to infection are coordinated through metabolic checkpoints, including AMP-activated protein kinase (AMPK), mechanistic target of rapamycin (mTOR), hypoxia-inducible factor-1α (HIF-1α), and mitochondrial bioenergetic pathways (78). Under physiological conditions, AMPK promotes energy homeostasis, autophagy, and antimicrobial responses, whereas mTOR supports anabolic growth programmes that can be exploited by both intracellular bacteria and respiratory viruses (79). During coinfection, these pathways become dysregulated through sustained inflammatory signaling and interferon-driven metabolic stress, producing heterogeneous activation states across immune-cell populations.

In macrophages, infection-induced shifts toward aerobic glycolysis and altered tricarboxylic acid cycle activity increase mitochondrial ROS production. While moderate ROS generation contributes to antimicrobial defense, excessive mitochondrial ROS and NADPH oxidase-derived ROS promote oxidative injury, cellular dysfunction, and inflammatory amplification (80). Respiratory viruses further intensify this metabolic–oxidative axis through IFN-I-dependent activation of mTORC1, stabilization of HIF-1α, and enhanced glycolytic flux, creating a Warburg-like metabolic state that favors viral replication while impairing effective antimycobacterial responses (81). Simultaneously, chronic inflammatory environments deplete antioxidant reserves and disrupt protective pathways involving metallothioneins and antioxidant enzymes, defects that are particularly evident in post-tuberculosis lung disease (82).

Metabolic state also influences tissue remodeling pathways. Dysregulated glucose and lipid metabolism are associated with increased expression of MMP-1, MMP-8, and MMP-9 and reduced tissue inhibitors of TIMPs, thereby promoting extracellular matrix degradation, cavitation, and fibrosis (83). Hypoxia and persistent HIF-1α signaling further reinforce inflammatory and matrix-remodeling programs, linking metabolic dysfunction to structural lung damage. These observations position metabolism upstream of oxidative stress and MMP activity, providing a mechanistic bridge between immune activation and the development of post-infectious pulmonary pathology (84, 85).

The therapeutic relevance of this axis has attracted increasing attention. AMPK agonists such as metformin restore metabolic homeostasis, enhance autophagic clearance, reduce excessive inflammation, and improve antimicrobial competence in experimental models of tuberculosis and viral infection. Collectively, these findings suggest that metabolic pathways are not merely consequences of infection but active regulators of disease outcome that may be exploited through host-directed therapeutic strategies (57).

### 7.2 Cell death as a bridge between metabolism and matrix injury

Cell death pathways function as critical effectors through which metabolic stress, oxidative injury, and inflammation are converted into tissue destruction. During an uncomplicated infection, apoptosis and autophagy contribute to pathogen containment by removing infected cells while limiting inflammatory damage (86). In *M. tuberculosis*–respiratory viral coinfection, however, these protective programmes are frequently subverted, shifting the balance toward lytic forms of cell death that promote bacterial dissemination and lung injury.

*M. tuberculosis* actively modulates host cell fate by inhibiting apoptosis and manipulating autophagic pathways, thereby enhancing intracellular survival. Respiratory viruses simultaneously engage extrinsic death pathways and inflammatory signaling cascades that further disrupt cellular homeostasis (87). Under conditions of sustained oxidative stress, mitochondrial dysfunction, and inflammasome activation, macrophages increasingly undergo pyroptosis, necroptosis, ferroptosis, and other forms of inflammatory cell death. These processes are characterized by membrane rupture, release of damage-associated molecular patterns, extracellular liberation of viable bacteria, and amplification of local inflammatory responses (88, 89).

Cell death, therefore, acts as a coupling node linking metabolic dysfunction to matrix injury. Oxidative stress promotes necrotic and pyroptotic cell death, which releases IL-1β, proteases, and matrix metalloproteinases into the granuloma microenvironment (83). Subsequent recruitment of neutrophils provides additional MMP-8, MMP-9, and ROS, establishing a feed-forward cycle in which inflammation drives metabolic stress, metabolic stress promotes lytic cell death, and cell death accelerates extracellular matrix degradation, cavitation, and lesion progression (86). Viral superinfection can intensify this process by increasing mitochondrial ROS production and shifting cell fate decisions away from apoptosis toward more inflammatory forms of death.

Importantly, cell death pathways also intersect with autophagy and mTOR signaling. Although autophagy can facilitate clearance of intracellular pathogens, prolonged metabolic dysregulation may convert protective autophagic responses into survival niches that favor pathogen persistence. Similarly, modulation of RIPK1/RIPK3/MLKL signaling and related necroptotic pathways has shown promise in preserving granuloma integrity and limiting tissue damage in experimental coinfection models (72, 81). These observations highlight cell death programs as mechanistic links between immune signaling, metabolic dysfunction, and pathological tissue remodeling, making them attractive targets for host-directed therapeutic intervention (57).

## 8 Adaptive immune dysregulation during coinfection

Adaptive immune responses during *M. tuberculosis*–respiratory viral coinfection reflect a dynamic recalibration of T−cell–derived cytokines and regulatory networks rather than a simple additive interference between two individual infections. In *M. tuberculosis*–respiratory−virus coinfection, Th1, Th17, regulatory T cells (Tregs), and exhausted T−cell compartments undergo characteristic shifts that rebalance protective containment against pathological inflammation. Understanding these changes in the context of TB stage and timing of viral exposure is critical for interpreting outcomes and designing immunomodulatory or vaccine strategies.

### 8.1 Th1 immune coordination and granuloma maintenance

Although Th1 responses remain central to mycobacterial containment, excessive or dysregulated IFN−γ signaling may also contribute to inflammatory tissue injury under specific coinfection contexts. In active TB and in experimental murine models, IFN−γ and TNF−α act in synergy to drive macrophage activation, induce reactive nitrogen intermediates, and support granuloma formation and maintenance (90). Human clinical and *in vitro* studies show that IFN−γ from CD4 T cells and γδ T cells licenses macrophages for phagosome maturation and restricts intracellular *M. tuberculosis* growth, thereby stabilizing granuloma architecture (91).

However, coinfection may distort this beneficial axis. Experimental murine models of TB-virus coinfection suggest that IFN−I−skewed environments impair dendritic−cell function, delaying IFN−γ and TNF production and weakening Th1 coordination (77). *In vitro* human systems further indicate that viral mimicry or IFN−I exposure reduces IL−12 production by dendritic cells, blunts Th1 differentiation, and dampens IFN−γ output from CD4 T cells changes that correlate with impaired granuloma maintenance in models and increased bacterial load in clinical cohorts (92). Concurrently, viral−mediated perturbation of chemokine receptors such as CCR2 can impair Th1−cell migration into granulomas, enhancing local bacterial dissemination without eliminating the global Th1 program. Thus, Th1 responses are necessary for granuloma stability but not sufficient for optimal protection when coinfection−driven dysregulation distorts IFN−γ timing, magnitude, and spatial coordination (85).

### 8.2 Th17 and mucosal inflammatory balance

IL−17–producing Th17 and γδ T cells sit at the core of a trade−off between mucosal antiviral defense and tissue−damaging hyperinflammation. In early respiratory virus infection, Th17−derived IL−17 supports neutrophil recruitment and epithelial barrier reinforcement, which can aid viral clearance at the airway mucosa (93). *In vitro* and murine studies of virus-induced pneumonia show that IL−17 amplifies neutrophil influx and enhances local antimicrobial peptide production, reinforcing mucosal defense without necessarily compromising antibacterial containment when inflammation remains transient (94).

In contrast, in *M. tuberculosis*–respiratory−viral coinfection, IL−1−β− and IL−6−rich microenvironments favor Th17 expansion and sustained IL−17 signaling, which shifts the balance toward tissue pathology (95). Experimental murine models of TB–viral superinfection show that IL−17−driven neutrophil recruitment correlates with alveolar and peribronchial inflammation, matrix remodeling, and cavity−like lesions, even when bacterial load is not yet maximal. Human histopathological and cytokine profiling studies in active TB and TB–COVID−19 coinfection indicate that elevated IL−17 and neutrophil−derived enzymes, such as elastase, ROS track with radiological cavitation and progressive lung damage, underscoring that mucosal IL−17–driven recruitment trades protective barrier defense for structural destruction when inflammation becomes chronic or amplified by viral inputs (93). In this context, early, controlled IL−17 signaling may preserve mucosal integrity, whereas persistent or coinfection−amplified Th17 activity risks eroding granuloma−adjacent structures and facilitating bacterial dissemination (96).

### 8.3 Regulatory T cells and immune suppression

Tregs and IL−10−producing cells function as major brakes on inflammatory responses, but in coinfection, their activity can tilt toward immune suppression and impaired bacterial control. In human TB and experimental murine models, IL−10 and Tregs limit collateral lung injury by counter−regulating Th1/Th17−driven cytokine storms, yet they also reduce IFN−γ and IL−12 production and constrain macrophage microbicidal activity (97). Recent reviews and clinical data highlight that IL−10 and Treg signatures are elevated in active TB and in HIV−TB coinfection, where they correlate with increased bacterial load and delayed sputum conversion, suggesting a double−edged role in immune regulation (98).

In *M. tuberculosis*–respiratory−virus coinfection, viral−induced IL−10 and Treg expansion can further impair Th1−mediated containment. *In vitro* and murine studies show that IL−10 suppresses IL−12 and TNF−α production from dendritic cells and macrophages, blunts antigen−specific T−cell proliferation, and promotes T−cell anergy effects that are particularly detrimental when co−localized with granulomas (97). Human clinical evidence from TB-COVID−19 cohorts suggests that SARS−CoV−2–driven IL−10 and regulatory signatures associate with diminished Th1 responses and prolonged illness, reinforcing the idea that Treg− and IL−10−mediated immune suppression may be protective against hyperinflammation but maladaptive when it undermines granuloma−supporting Th1 function (98). Thus, during coinfection, the balance between regulatory suppression and effective immunity becomes a key determinant of whether granulomas remain stable or drift toward dissemination−prone states.

### 8.4 T−cell exhaustion and checkpoint signaling

T−cell exhaustion and checkpoint signaling (PD−1, TIM−3, etc.) represent a third layer of adaptive dysregulation that links chronic TB, recurrent viral exposure, and inflammatory persistence. In chronic TB and in experimental murine models, persistent antigen exposure leads to progressive CD4 and CD8 T−cell exhaustion, marked by co−upregulation of PD−1 and TIM−3, reduced IFN−γ and TNF production, and impaired proliferative capacity (99). Recent single−cell and *in vivo* murine work indicates that the emergence of PD−1^+^ TIM−3**^+^**CD4 T cells correlates with bacterial recrudescence and disease progression, raising the possibility that exhaustion is not merely a marker of chronicity but a functional driver of impaired bacterial control (92).

In coinfection contexts, recurrent or chronic viral exposure can amplify this exhaustion program. *In vitro* and clinical studies in HIV−TB and other co−infection systems show that PD−1 and TIM−3 blockade can partially restore IFN−γ production and macrophage control over *M. tuberculosis*, at least in combination with antimicrobial therapy (98). Limited human data from TB–viral−coinfection cohorts, including post−COVID−19 TB reactivation, suggest that persistent expression of checkpoint molecules on T cells coincides with prolonged inflammatory signatures and delayed recovery, consistent with a model in which chronic immune activation and exhaustion co−exist and mutually reinforce bacterial persistence. Therefore, T−cell exhaustion ties together chronic TB, repeated viral antigenic exposure, and sustained inflammatory environments, positioning PD−1/TIM−3 and related pathways as potential targets for restoring coordinated T−cell–mediated control without unleashing uncontrolled pathology (99).

Collectively, adaptive immune responses during coinfection appear to reflect a dynamic balance between protective containment and pathological inflammation, with disease outcomes likely shaped by the timing, magnitude, and coordination of T−cell−mediated immunity. TB is not a single immunological state, and its interaction with respiratory viruses remodulates Th1/Th17/Treg checkpoints uniquely across latent, subclinical, active, and reactivation states (64). Experimental murine models strongly support the idea that IFN−γ–driven macrophage activation and granuloma stability co−exist with risk of inflammatory injury when IFN−γ is dysregulated, while human clinical and *in vitro* evidence indicates that viral−induced Th17 skewing, IL−10/Treg expansion, and PD−1/TIM−3–driven exhaustion can all shift this balance toward impaired containment or tissue destruction depending on context (77). Explicitly distinguishing these evidentiary tiers-murine, *in vitro*, organoid, and human- strengthens the mechanistic narrative and clarifies where the field can leverage adjuvant−driven immune polarization, checkpoint modulation, or cytokine−targeted interventions to restore a more protective adaptive equilibrium (95, 99).

## 9 Host-directed therapeutic opportunities: timing, endotypes, and trade-offs

### 9.1 Immunometabolic therapies

Metformin and statins exemplify immunometabolic HDTs that rewire macrophage metabolism, mitochondrial function, and redox balance, with important benefits and trade−offs. Metformin activates AMPK, which shifts macrophages toward oxidative phosphorylation and promotes iron restriction and mitochondrial ROS production, creating a hostile intracellular environment for *M. tuberculosis* (100, 101). Experimental models show that metformin−induced autophagy, AMPK−dependent signaling, and altered mitochondrial ROS can reduce intracellular bacterial load and dampen inflammatory myeloid recruitment. In murine and quantitative systems−pharmacology frameworks, metformin adjunctively delays disease progression in diabetic TB models, suggesting that immunometabolic rewiring can complement antibiotics when the overall bacterial burden and extracellular−to−intracellular ratio are low (102).

Statins exert pleiotropic effects on cholesterol and isoprenoid metabolism, reduce ROS, and modulate mitochondrial and inflammatory signaling in macrophages. Experimental models indicate that statins can attenuate excessive TNF−α and IL−1β production while preserving macrophage antimicrobial function, which may limit granuloma−associated tissue damage without fully suppressing bacterial containment (103). However, despite promising immunomodulatory effects in experimental models, clinical outcomes have remained variable across heterogeneous patient populations: recent clinical trials of adjunctive metformin added to standard TB treatment report no significant reduction in time to sputum conversion, and early large−scale statin−TB trials have shown inconsistent effects on radiological and microbiological outcomes (102). These limitations underscore that immunometabolic therapies are sensitive to underlying metabolic and immune endotypes, and that their benefit may be confined to specific windows, such as early−stage or comorbid−diabetes cohorts, rather than universally applicable.

### 9.2 Cytokine and interferon modulation

Cytokine and interferon−modulating agents must be evaluated in the context of the dominant phase in coinfection: is the system prioritizing antiviral control or mycobacterial containment? Interventions targeting IFN−I signaling may exert divergent effects depending on whether antiviral control or mycobacterial containment predominates during a given phase of coinfection, a concept now supported by HDT and cytokine−blockade experiments in TB–virus models. In early viral infection, transient or localized IFN−I blockade can reduce viral−driven immunopathology and preserve IL−1/TNF−dependent antibacterial responses, whereas in later stages, suppressing IFN−I too broadly may impair viral clearance and release immune brakes on bacterial replication (100, 104).

Targeting the IL−1 axis also carries strong timing dependence. In experimental TB models, enhancing IL−1 signaling improves granuloma formation, Th1 priming, and IL−13−dependent granuloma maturation, whereas IL−1 blockade or receptor−deficient mice show impaired bacterial control and dysregulated granulomas. In coinfection, IL−1−modulating strategies which are, anakinra or IL−1R antagonists, may reduce tissue damage but risk weakening antibacterial containment if applied during active TB or early viral clearance, highlighting a trade−off between inflammation control and microbial control (105).

TNF−modulating agents and corticosteroids illustrate parallel principles. In active TB and in TB−drug−reaction or TB−IRIS settings, short−course corticosteroids and TNF dampening can reduce life−threatening hyperinflammation, yet in latent or early−stage TB, they increase the risk of reactivation and dissemination, underscoring that TNF’s role shifts from protective granuloma maintenance to pathological driver depending on stage and host immune status (90). Thus, cytokine and IFN−directed therapies must be titrated by phase (viral−dominant vs. bacterial−dominant) and host endotype (e.g., HIV−coinfected vs. immunocompetent) (91).

### 9.3 Inflammasome and inflammatory targeting

Inflammasome−targeted HDTs aim to reduce tissue injury driven by NLRP3, IL−1β, and pyroptosis while preserving sufficient antibacterial signaling. Experimental models show that *M. tuberculosis* activates NLRP3 in ESX−1–dependent and independent ways, driving IL−1β and IL−18 release and pyroptotic cell death, which can amplify local inflammation and contribute to granuloma necrosis (106). *In vitro* and murine studies with selective NLRP3 inhibitors such as MCC−950 demonstrate that abrogating inflammasome signaling reduces IL−1β/IL−18 secretion and cell−death−associated tissue damage; however, genetic deletion studies indicate that NLRP3 and caspase−1 are not strictly required for bacterial control, whereas ASC−dependent signaling is more tightly linked to granuloma integrity (105).

These findings suggest a therapeutic window where NLRP3−directed inhibitors can reduce tissue injury without broadly impairing mycobacterial containment but also highlight a risk: excessive suppression of inflammasome activity may blunt IL−1R1−dependent T−cell priming and granuloma stabilization, increasing susceptibility to bacterial dissemination, particularly in chronic or reactivation−prone settings (104). In coinfection, inflammasome−targeted HDTs may be most appropriate in hyperinflammatory phases (e.g., active TB with severe viral−superimposed lung injury), where dampening pyroptosis and IL−1β can limit immunopathology, but they should be avoided in early containment or latent−TB−recovery windows where inflammasome−driven priming and local containment are critical (103).

### 9.4 Therapeutic windowing and stage-specific interventions

Therapeutic windowing is where mechanism−based HDT design becomes clinically realistic. Below is a stage−specific, timing−aware framework derived from murine, *in vitro*, and early−phase human data; these remain hypothesis−driven and not yet fully validated in randomized trials (102). In the acute viral phase, the goal is antiviral−centric modulation, ideally by fine−tuning IFN−I signaling and cautiously restraining IL−1/IL−18 and TNF−α when they threaten severe immunopathology; suppressing these pathways too broadly may prolong viral replication and later destabilize mycobacterial containment (104). In latent TB, the priority shifts to immune stabilization, where mild immunometabolic support (for example, AMPK−targeting agents such as metformin) or controlled IL−1/TNF priming may strengthen granuloma integrity and delay reactivation, while strong immunosuppressants such as long−term TNF blockers clearly increase reactivation risk (103). In active TB, the focus becomes limiting inflammation and tissue injury, with short−course corticosteroids or cautious NLRP3/IL−1–targeted strategies used to reduce necrotizing and fibrotic lung damage without fully dismantling granuloma−supporting cytokine networks (90). Finally, in the recovery or post−TB phase, the window opens for tissue−repair−oriented interventions, for example, adjunctive metformin or antioxidants, aimed at improving lung−function recovery and reducing post−infectious fibrosis rather than directly modulating antimicrobial immunity.

### 9.5 Limitations and failed HDTs

Despite strong mechanistic rationales, many HDTs have yielded heterogeneous or failed outcomes in clinical trials, exposing critical limitations in design and host stratification (103). Experimental murine and *in vitro* systems show that adjunctive metformin or statin−based immunometabolic modulation can reduce bacterial load or inflammatory myeloid recruitment, yet human trials often report no significant improvement in microbiological or radiological endpoints, likely due to patient heterogeneity in age, comorbidities, immune endotypes, and disease stage (100). Biomarker limitations further complicate interpretation: current markers poorly distinguish patients in whom IFN−I or IL−1 blockade will be protective from those in whom the same pathway is essential for containment, leading to underpowered or “diluted” trial signals.

Immune over−suppression represents a recurring risk: TNF−targeted strategies that are effective in severe hyperinflammation can trigger reactivation TB in latent or early−stage disease, and inflammasome− or IL−1−targeted interventions that reduce tissue injury may simultaneously impair granuloma−supporting T−cell responses (105). These inconsistencies underscore that, to date, HDT translation has been mechanistically plausible but clinically precarious, with outcomes highly dependent on matching drug mechanism, immune window, and host endotype rather than applying blanket adjunctive regimens (106). Future strategies must therefore integrate biomarker−driven staging (latent, subclinical, active, reactivation) and define narrow, phase−specific therapeutic windows to avoid the pitfalls of immune−suppressing therapies that failed to distinguish protection from pathology.

### 9.6 Immune endotypes and precision HDT framework in coinfection

Host-directed therapy outcomes in *M. tuberculosis*–respiratory virus coinfection are highly variable because immune responses are not uniform across patients or disease stages. This variability can be better understood through the concept of immune endotypes, stable yet dynamic patterns of immune activity that integrate cytokine balance, metabolic state, and T-cell functionality (107). Broadly, coinfected hosts can be stratified into three overlapping endotypes: a Th1-dominant containment profile with effective granuloma control, a neutrophilic–hyperinflammatory profile associated with tissue damage and cavitation, and a regulatory/exhaustion profile characterized by impaired effector responses and immune suppression. Respiratory viruses further reshape these states, particularly through IFN-I–driven suppression of Th1 signaling, metabolic reprogramming toward glycolysis, and amplification of inflammasome activity (108).

Within this framework, HDT efficacy is inherently context-dependent. Anti-inflammatory strategies such as IL-1 or NLRP3 modulation may benefit hyperinflammatory phenotypes but risk impairing bacterial containment, while immune-revitalizing approaches may be required in exhaustion-dominant states but could exacerbate pathology if misapplied (109). Similarly, immunometabolic interventions such as AMPK activation may be beneficial only within specific phases of infection and host metabolic states. These interactions explain why many HDTs show strong mechanistic promise but inconsistent clinical outcomes: they are typically applied without immune stratification (110). A precision approach, therefore, requires aligning therapy with three variables: immune endotype, infection stage, and dominant pathological process (viral injury, bacterial persistence, or tissue damage).

In this model, immune endotypes provide a dynamic framework for selecting and timing HDTs, shifting treatment design from uniform immunomodulation toward stratified, phase-specific immune recalibration in coinfection.

**Table 2** summarizes pathway-aligned candidates, highlighting the need for precision timing to avoid compromising antiviral control or granuloma stability.

**Table 2:** Candidate host-directed therapy (HDT) targets in *M. tuberculosis*–respiratory virus coinfection: mechanisms, evidence, and timing considerations.

Target node/pathway	Candidate intervention(s)	Mechanistic rationale in coinfection	Evidence signal	Key risks/cautions	Timing considerations
Type I IFN axis (IFNAR–ISG dominance)	Transient IFNAR pathway modulation (conceptual; investigational)	Virus-augmented IFN-I can impede Th1 pulmonary influx and suppress protective antimycobacterial immunity.	IFN-I-dependent impediment of Th1 influx worsens TB immunopathogenesis in viral coinfection models (18).	May compromise antiviral defence if applied too early/too broadly.	Consider only after the viral peak and in IFN-high pathology endotypes.
PRR convergence (STING/TBK1–IRF3)	STING/TBK1-axis tuning (experimental)	Shared nucleic-acid sensing amplifies IFN-I and remodels DC programming; potential checkpoint to rebalance outputs.	*M. tuberculosis* induces Irg1 via coordinated TLR2 and STING/IFNAR signaling, illustrating actionable crosstalk (134).	Broad suppression risks weakening both antiviral and antibacterial sensing.	Stage- and compartment-specific modulation; requires preclinical validation.
Immunometabolism (AMPK activation/mTOR restraint)	Metformin (AMPK agonism); mTOR modulation (conceptual)	Counteracts virus-driven glycolytic bias; promotes autophagy and energy homeostasis; may improve intracellular control and limit inflammation.	*M. tuberculosis* restrains glycolysis via miR-21/PFK-M; metformin adjunct trial shows early sputum conversion benefit (78, 135).	Host heterogeneity (diabetes, malnutrition) may alter benefit; avoid overgeneralization.	Most plausible in subacute/recovery phase to reduce persistent inflammation and support TB therapy.
Inflammasome (NLRP3–caspase-1–GSDMD)	NLRP3 inhibitors (e.g., MCC950; experimental); IL-1 pathway modulation (select contexts)	*M. tuberculosis* membrane damage and viral K+ efflux/viroporins converge on NLRP3 → pyroptosis and lung injury.	*M. tuberculosis* membrane damage activates NLRP3/pyroptosis; SARS−CoV−2 ORF3a triggers NLRP3 (72–74).	IL−1 can be protective in TB; over-inhibition may impair containment.	Target only pathology-dominant endotypes; late viral phase/hyperinflammation with careful monitoring.
Checkpoint pathways (PD-1)	Avoid PD−1 blockade as TB HDT (oncology indication only)	Checkpoint disruption can destabilize granuloma control and precipitate TB worsening/reactivation.	Coinfection models emphasize the importance of coordinated Th1 trafficking; checkpoint disruption is high-risk (18).	Risk of TB reactivation/worsening immunopathology.	Not recommended as TB coinfection HDT; if unavoidable, enforce TB screening/monitoring.
Broad cytokine suppression (e.g., TNF blockade)	Generally, avoid in TB-risk settings	TNF supports granuloma integrity; suppression can cause dissemination despite reducing inflammation.	Clinical experience supports the essential role of TNF in TB containment (contextual).	High TB reactivation/dissemination risk.	Not a coinfection HDT strategy; reserve only for compelling indications with TB precautions.

## 10 Biomarkers and immune stratification in *M. tuberculosis*-respiratory virus coinfection

Translating mechanistic insights into clinical practice requires a biomarker framework that links molecular pathways to host immune endotypes, disease stage, and clinical outcomes in *M. tuberculosis*–respiratory viral coinfection. The most promising molecular targets for diagnosis and prognosis converge on a limited set of readouts that reflect IFN signatures, oxidative stress and ROS, and MMP/TIMP balance, each with clear prognostic relevance and potential to guide precision therapy.

Interferon signatures are among the most robust and actionable biomarkers. Elevated IFN−I−related transcripts, including IFN−β, ISG15, MX1, STAT1/IRF7 upregulation, IFNAR signaling readouts, and concomitant suppression of IFN−γ/STAT1 activity, indicate a Type I interferon–dominant state linked to impaired bacterial control and higher TB reactivation risk (66). In TB–respiratory viral coinfection, these IFN signatures correlate with hyperinflammation, increased lung pathology, and adverse clinical trajectories, and they can be used to stratify patients into endotypes that may benefit from IFN−I modulation versus those in whom IFN−I suppression could compromise antiviral control (98).

Markers of oxidative stress and mitochondrial dysfunction provide complementary prognostic information. Elevated markers of systemic oxidative stress, mitochondrial ROS, and impaired antioxidant capacity, for example, reduced glutathione, altered metallothioneins, increased lipid peroxidation products, are associated with more severe disease, greater epithelial injury, and poorer lung−function outcomes in TB and post−TB populations (80). In coinfection, ROS markers may indicate hosts in whom inflammation is being efficiently converted into tissue injury, identifying individuals who might benefit from antioxidant or metabolic−targeted host−directed therapies.

The MMP/TIMP axis directly links inflammation to matrix remodeling and structural lung disease. Elevated MMP−1, MMP−8, and MMP−9 in serum or bronchoalveolar lavage, together with reduced TIMP levels, associate with cavity formation, radiological severity, fibrosis, and impaired lung function. In coinfection, MMP/TIMP signatures can stratify patients into those at high risk for cavitation and progressive lung damage versus those with more contained disease, and they may guide the use of anti−inflammatory or MMP−targeted interventions (57, 111).

Together, these molecular readouts can be integrated into host immune endotypes- high−Th1 or IFN−γ−biased, high−inflammatory or neutrophilic, and high−regulatory/exhaustion−prone, that capture distinct patterns of immune dysregulation, disease risk, and therapeutic response (112). Such endotype−informed stratification has clear prognostic relevance: it enables earlier identification of patients at risk for reactivation, severe lung injury, and fibrosis, and supports precision approaches that match host−directed therapies to the patient’s immune and metabolic state rather than applying uniform adjunctive regimens (113, 114).

Addressing these gaps will require longitudinal human cohorts coupled to time-resolved experimental systems and spatial multi-omics, enabling rational design of host-directed therapies that restore protective Th1 immunity while avoiding loss of antiviral control (**Table 3**).

**Table 3:** Critical knowledge gaps and priority research directions in *M. tuberculosis*- respiratory virus coinfection (with in-text references).

Domain	Critical knowledge gap	Priority approaches	Key translational endpoints	Key refs
Infection order & timing	How does virus→*M. tuberculosis* vs *M. tuberculosis*→virus sequence and interval alter IFN-I/Th1 balance and outcomes?	Sequential infection models; time-resolved sampling; IFNAR/TBK1 perturbation windows.	IFN-I/IFN-γ ratio; Th1 influx; bacterial burden kinetics.	(18, 21)
PRR convergence (TLR–RLR–STING)	Which PRR nodes drive synergy vs antagonism and in which compartments (airway vs granuloma)?	CRISPR/pathway inhibition; phosphoproteomics (TBK1/IKK/IRF3/NF-κB); spatial profiling.	ISG score; IL-12/DC maturation; IRF3 phosphorylation.	(18, 134)
Immunometabolism (mTOR–AMPK–HIF-1α)	Cell-type-specific metabolic states during coinfection and how they shape killing vs pathology.	Isotope tracing; Seahorse assays; conditional mTOR/AMPK models; hypoxia mapping.	Lactate; autophagy flux; mitochondrial stress markers; culture conversion.	(78, 135)
Epigenetic imprinting/trained immunity	Do post-viral epigenetic ‘immune scars’ in myeloid progenitors increase TB reactivation risk?	Longitudinal cohorts; scRNA/scATAC; chromatin mark profiling; progenitor tracking.	Persistent ISG chromatin accessibility; inflammatory recall signatures.	(21, 136)
Inflammasome & cell death	Relative roles of NLRP3 vs AIM2 and pyroptosis vs apoptosis/necroptosis in dissemination and lung injury.	Genetic dissection; gasdermin D cleavage assays; live-cell fate imaging; ORF3a/NLRP3 studies.	IL-1β/IL-18; gasdermin D fragments; epithelial injury/necrosis markers.	(72–74)
Adaptive immunity/exhaustion & granuloma stability	When does PD-1–linked exhaustion reflect protective restraint vs failed control in coinfection?	Paired TCR-seq+phenotyping; antigen presentation assays; spatial granuloma immune mapping.	PD-1/TIGIT expression; Th1 polyfunctionality; granuloma architecture metrics.	(18, 21)

The overlapping clinical presentations of TB and respiratory viral infections further complicate timely diagnosis, particularly in low- and middle-income countries where healthcare resources are constrained. Consequently, coinfected individuals face increased morbidity, mortality, and risk of ongoing transmission.

Future efforts should focus on early detection and individualized management of coinfections. Understanding the molecular signaling that drives the modulation of the immune response may lead to several important solutions that will enhance the diagnosis and prognosis of patients. This includes the possible design of advanced multiplex diagnostics and biomarker-guided approaches essential to enable rapid and accurate identification of overlapping infections. Host-directed therapies that restore immune balance, alongside tailored antimicrobial regimens, offer promise in mitigating severe disease outcomes. This will, in the future, strengthen integrated surveillance systems and multidisciplinary care networks and support coordinated responses during epidemics and pandemics. Additionally, sustained investment in TB immunology research is critical to unravelling the molecular and immunological pathways underlying coinfections, identifying novel therapeutic targets, and informing public health interventions. Collectively, these strategies will enhance patient outcomes, particularly in resource-limited settings, and contribute to global TB control in the era of respiratory viral epidemics.

## 11 Integrated systems model and future recommendations

This review proposes an integrated systems model of *M. tuberculosis*–respiratory virus coinfection in which immune rewiring, temporal dynamics, and pathway cross−talk converge to drive tissue injury and shape clinical outcomes. Rather than viewing viral and bacterial infections as separate entities superimposed on the same lung, the model frames coinfection as a dynamic, stage−dependent reconfiguration of host networks that begins at pathogen sensing and propagates through interferon, inflammasome, metabolic, and cell−death modules to alter granuloma equilibrium and lung architecture.

**Figure 2:**
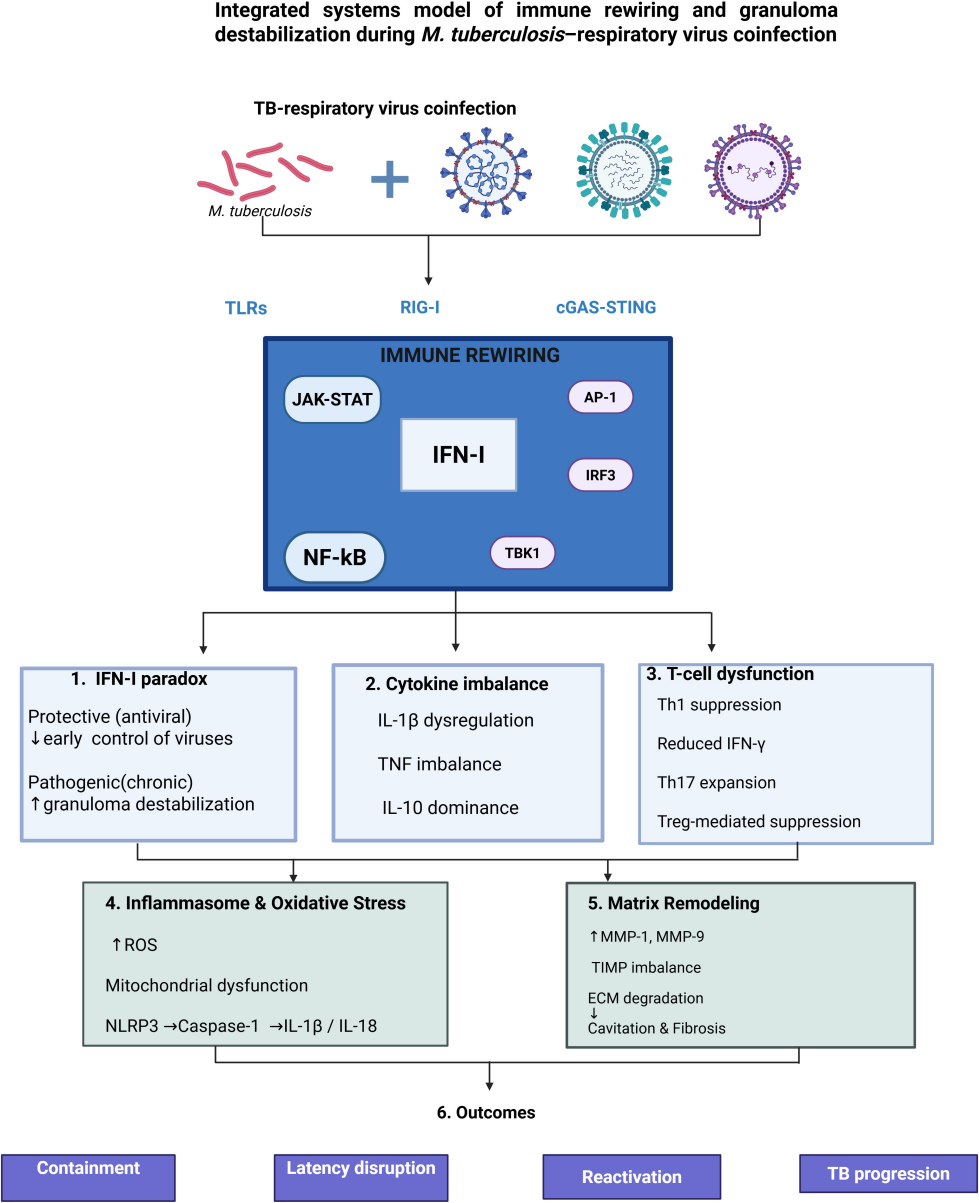
Respiratory viral coinfection triggers a convergent immunoregulatory hub centered on type I interferons, NF-κB, JAK–STAT, and IRF3/IRF7 pathways, which coordinately reshape host responses. This integrated signaling axis underlies the IFN-I paradox, skews cytokine networks, and drives T-cell dysfunction, thereby fostering inflammasome activation, redox imbalance, and aberrant matrix remodeling. Consequent downstream events—including excessive ROS generation, NLRP3 inflammasome engagement, mitochondrial injury, and dysregulated MMP activity—promote granuloma destabilization, tissue injury, cavitation, and fibrotic remodeling. Depending on the temporal sequence of infection, the host immune endotype, and the underlying stage of TB disease, these processes may alternatively preserve granuloma integrity or disrupt latency, precipitating reactivation, progressive disease, or severe lung pathology. Together, these context-dependent outcomes expose rational windows for biomarker-informed risk stratification and host-directed therapeutic targeting.

According to **Figure 2**, at the core of the model is immune rewiring, initiated by overlapping pathogen recognition pathways (TLR, RIG−I, cGAS–STING) that converge on IFN-I, NF−κB, and AP−1 signaling. Viral infection can transiently or persistently skew these pathways, suppressing Th1/IFN−γ–driven macrophage activation, amplifying Th17/neutrophilic inflammation, and engaging regulatory or exhausted T−cell states. This rewiring is not static; it exhibits temporal dynamics that depend on pathogen order, viral burden, and TB stage. An early, transient IFN−I pulse may be largely protective, whereas sustained IFN−I or late viral superinfection can destabilize granulomas, promote reactivation, and accelerate progression to active disease.

Pathway cross−talk between interferon, inflammasome, cytokine, and metabolic networks amplifies these effects. IFN−I intersects with NLRP3/IL−1β and ROS production to shape pyroptosis and necroptosis, while metabolic reprogramming (glycolysis, AMPK/mTOR, mitochondrial dysfunction) sets the oxidative stress tone and modulates MMP expression. In turn, oxidative stress and MMP/TIMP imbalance convert inflammatory signals into epithelial damage, cavitation, and fibrosis. Thus, tissue injury emerges not from a single cytokine or pathway but from the integrated output of multiple interconnected modules.

The model also defines therapeutic windows that are stage− and endotype−specific. In the acute viral phase, interventions may focus on antiviral−centric modulation and careful IFN−I tuning; in latent or subclinical TB, the goal shifts to immune stabilization; in active TB, the focus becomes inflammation and tissue−injury control; and in the recovery phase, therapies may target fibrosis and lung−function repair. Precision host−directed therapies that match mechanism to host immune endotype and timing are more likely to succeed than blanket adjunctive regimens.

Future directions include defining quantitative biomarker signatures that capture IFN, ROS, and MMP/TIMP readouts; mapping single−cell and transcriptomic endotypes across TB stages and coinfection; developing systems−level computational models that integrate these layers to predict outcomes; and testing timing−aware, endotype−stratified interventions in clinical trials. By integrating viral sensing, immune rewiring, granuloma biology, oxidative stress, metabolism, and cell death into a unified framework, this model provides a mechanistic foundation for precision approaches to TB–respiratory virus coinfection.

## 12 Conclusion

Coinfections of *M. tuberculosis* with respiratory viruses, including SARS−CoV−2, influenza A/B, RSV, metapneumovirus, parainfluenza, rhinovirus, adenovirus, and bocavirus, represent a complex clinical and public health challenge that cuts across epidemiology, immunology, and precision therapeutics. These coinfections exacerbate disease severity by dysregulating host immune responses, impairing Th1−mediated protection, amplifying Th17/neutrophilic inflammation, and promoting hyperinflammation, oxidative stress, MMP−driven matrix remodeling, and accelerated TB progression. Crucially, TB is not a single immunological state: latent, subclinical, active, and reactivation disease each support distinct immune landscapes that respond differently to viral superimposition, and outcomes depend on timing (infection order and stage), context (viral burden and host immune endotype), and compartmentalization (airway versus granuloma).

This manuscript synthesizes current knowledge into an integrated systems framework that links viral sensing (TLR, RIG−I, cGAS–STING) to immune rewiring (IFN−I skewing, Th1/Th17/Treg imbalance, T−cell exhaustion), granuloma destabilization, and tissue injury through oxidative stress, metabolism, and MMP/TIMP pathways. We emphasize that the same molecular perturbation can be protective or pathogenic depending on the temporal window and host endotype, underscoring the need for stage−specific, endotype−informed host−directed therapies and timing−aware clinical strategies. Priority knowledge gaps that currently limit mechanistic integration and translation include the need for quantitative biomarker signatures (IFN, ROS, MMP/TIMP), single−cell and transcriptomic mapping of immune endotypes across TB stages, systems−level computational models of coinfection dynamics, and clinical trials that stratify patients by timing, stage, and immune phenotype. Addressing these gaps will enable precision approaches that improve diagnosis, prognosis, and therapeutic management of *M. tuberculosis*–respiratory virus coinfection in vulnerable populations.
